# A Cross-Cultural Analysis of the Influence of Timbre on Affect Perception in Western Classical Music and Chinese Music Traditions

**DOI:** 10.3389/fpsyg.2021.732865

**Published:** 2021-09-29

**Authors:** Xin Wang, Yujia Wei, Lena Heng, Stephen McAdams

**Affiliations:** ^1^School of Music and Recording Art, Communication University of China, Beijing, China; ^2^Schulich School of Music, McGill University, Montreal, QC, Canada

**Keywords:** timbre, affect perception, cross-cultural, valence, tension arousal, energy arousal

## Abstract

Timbre is one of the psychophysical cues that has a great impact on affect perception, although, it has not been the subject of much cross-cultural research. Our aim is to investigate the influence of timbre on the perception of affect conveyed by Western and Chinese classical music using a cross-cultural approach. Four listener groups (Western musicians, Western nonmusicians, Chinese musicians, and Chinese nonmusicians; 40 per group) were presented with 48 musical excerpts, which included two musical excerpts (one piece of Chinese and one piece of Western classical music) per affect quadrant from the valence-arousal space, representing angry, happy, peaceful, and sad emotions and played with six different instruments (*erhu*, *dizi*, *pipa*, violin, flute, and guitar). Participants reported ratings of valence, tension arousal, energy arousal, preference, and familiarity on continuous scales ranging from 1 to 9. ANOVA reveals that participants’ cultural backgrounds have a greater impact on affect perception than their musical backgrounds, and musicians more clearly distinguish between a perceived measure (valence) and a felt measure (preference) than do nonmusicians. We applied linear partial least squares regression to explore the relation between affect perception and acoustic features. The results show that the important acoustic features for valence and energy arousal are similar, which are related mostly to spectral variation, the shape of the temporal envelope, and the dynamic range. The important acoustic features for tension arousal describe the shape of the spectral envelope, noisiness, and the shape of the temporal envelope. The explanation for the similarity of perceived affect ratings between instruments is the similar acoustic features that were caused by the physical characteristics of specific instruments and performing techniques.

## Introduction

Music is an important medium of emotional communication. The expression and perception of musical emotion are related to psychophysical and cultural cues (Balkwill and Thompson, 1999; Balkwill et al., 2004). Psychophysical cues refer to musical elements that are usually represented by designations in conventional musical notation, such as pitch, dynamics, tempo, rhythm, instrument (timbre), mode, and harmony. Some psychophysical cues are universal and the basis for emotional communication between individuals with different cultural backgrounds. Cultural cues refer to musical expressions formed during the development of specific musical cultures, such as the way the note is terminated and specific articulation (Thompson and Balkwill, 2010). People need to learn special expression rules in a long-term listening environment to build a relationship between expression rules and affect. The similarities and differences in how psychophysical and cultural cues influence perceived affect across cultures is still a topic of concern in academia (Fritz et al., 2009).

Researchers usually use affective models to measure perceived affect through self-report. There are two main affective models: the categorical and dimensional models. The dimensional model can describe continuous perceptual levels and is convenient for establishing an association between affect and acoustic features through regression analysis or other methods. The most notable dimensional model, based on circumplex model of Russell (1980), combines two core dimensions, valence, and arousal (Schubert, 1999). The other two-dimensional models with higher citation rates are the positive and negative affective model (Watson et al., 1988) and the tension arousal (tension-relaxation) and energy arousal (awake-tiredness) model (Thayer, 1986). Adding a third dimension has been proposed over the years due to drawbacks with the two-dimensional model. In the field of music and emotion research, the most convincing three-dimensional model is the combination of two-dimensional models of Russell (1980) and Thayer (1986), which includes valence, tension arousal, and energy arousal. Schimmack and Grob (2000) demonstrate that the two-dimensional model does not adequately capture the structure of affective data in their study and that the three-dimensional model fares better. They propose on the basis of neurophysiological studies that the degree of tension arousal reflects activity of the activation system, whereas the degree of energy arousal reflects activity of the arousal system. Since then, many researchers have confirmed this conclusion and applied this model to music affective perception experiments (Ilie and Thompson, 2006; Vuoskoski and Eerola, 2010; Zentner and Eerola, 2010; McAdams et al., 2017). The three-dimensional model of affect was adopted in this paper.

Since the 1930s, researchers have begun to explore the association between psychophysical cues and affect perception. Psychophysical cues have mainly focused on perceptual elements including loudness (Leman et al., 2005) and roughness (Farbood and Price, 2014), and structural elements including mode (Fang et al., 2017), harmony (Gabrielsson and Lindström, 2010), and tempo (Baraldi et al., 2006; Zhang and Pan, 2017). Although, musicians choose different instruments to express specific affects, little research has been conducted on the influence of timbre on affect perception until the 1990s. Researchers have confirmed that timbre is correlated with perceived discrete affects. In a study by Behrens and Green (1993), results showed that participants could identify three types of affective content in solo improvisations performed in four different timbres, and the judgment depended not only on timbre but also on the affect expressed. Wu et al. (2014) compared different Western sustaining instruments in their expression of eight affects using a paired-comparison method. The results indicated that the violin, trumpet, and clarinet were the most suitable for expressing happy emotions, whereas horns and flutes were more likely to convey sad emotions. Their paper also confirmed that brightness, attack time, and odd-even harmonic energy ratio were highly correlated with affect perception. A subsequent study conducted in Western non-sustaining instruments extended this work and found that the guitar, harp, and plucked violin were highly related to negative affect. The decay slope and density of harmonics were significant timbral features of affective perception of Western non-sustaining instruments (Chau et al., 2015). The relationship between timbre and dimensional affect first attracted attention in 2012. Eerola et al. (2012) used a three-dimensional affect model and emotional dissimilarity ratings to collect the affect ratings of isolated instrument sounds with the same duration, pitch, and dynamics, and explored the relationship between acoustic features related to timbre and perceived affect ratings. Their research indicated that valence and energy arousal could be predicted by linear combinations of a few acoustic features. The role of timbre and pitch register in perceived affect ratings has been examined by McAdams et al. (2017) in an extension of the Eerola et al. (2012) study. They used 137 Western musical tones played at pitch class D# across each instrument’s entire pitch range at a forte dynamic level and found that various timbral features were important for explaining the three perceived affect ratings. Furthermore, each affect dimension was carried by a distinct set of timbral features. Until now, most research has focused on Western music and affect perception in Western participants. Little research has been conducted on affect perception with non-Western instruments. In a study related to Chinese culture, Liu and Liu (2011) used the *zheng* (plucked zither) and *xun* (vessel flute similar to the ocarina) to play Chinese classical music and explored the relationship between affect and physiological indicators. They found that music with different timbres could successfully induce different affects.

The cue-redundancy model was proposed based on the relationship between music and emotion in cross-cultural research (Balkwill and Thompson, 1999). Many cross-cultural studies have confirmed that participants were sensitive to the intended emotion aroused by unfamiliar music through attending to psychophysical cues (Argstatter, 2015; Cowen et al., 2020) such as tempo and rhythm (Balkwill et al., 2004; Fritz et al., 2009; Zacharopoulou and Kyriakidou, 2009; Laukka et al., 2013; Midya et al., 2019), complexity (Balkwill et al., 2004), harmonic dissonance (Athanasopoulos et al., 2021; Lahdelma et al., 2021), and tonality (Laukka et al., 2013; Egermann et al., 2015; Raman and Dowling, 2017; Midya et al., 2019). Timbre is one of the psychophysical cues that has a great impact on affect perception in cross-cultural research, although not much research has been conducted on this. Hu and Yang (2017) explored which acoustic features predicted perceived affect ratings based on an affect regression model for Western and Chinese pop songs. Their results revealed that timbre features worked well for both valence and arousal prediction. Heng (2018) studied how timbre functioned in communicating affects in Western classical and Chinese music traditions. Their research indicated that participants trained in the different musical cultures identified the intended emotion significantly differently, and Chinese participants performed more accurately on the judgments of affects conveyed by the performances of both the Chinese and Western instrumentalists. If the stimuli and participants stemmed from the same culture, participants found it easier to decode musical emotion because they could draw from both psychophysical and cultural cues. This phenomenon is known as the in-group advantage, which is associated with in-group familiarity with a given cultural and social background (Elfenbein and Ambady, 2002; Argstatter, 2015).

The current study aims to extend previous research investigating the influence of timbre on the perception of affect conveyed by Western and Chinese classical music using a cross-cultural approach by answering the following four research questions:

*1. Which musical instruments convey similar perceptions of affect: instruments from the same category or instruments from the same culture?*

Six musical instruments were included in this experiment, which were a Chinese bowed chordophone – *erhu*, a Western bowed chordophone – violin, a Chinese plucked chordophone – *pipa*, a Western plucked chordophone – guitar, a Chinese aerophone – *dizi,* and a Western aerophone – flute. To comprehensively examine the differences in the perception of four types of affect conveyed by these six instruments, participants from different cultural and musical backgrounds, including Western and Chinese classical music, were involved in this experiment.

*2. Do participants’ cultural or musical backgrounds have a greater impact on affect perception?*

Four LGs were included: listeners trained in Western classical music from Canada (hereafter termed Western musicians), listeners trained in Chinese classical music from China (hereafter termed Chinese musicians), nonmusicians from Canada (hereafter termed Western nonmusicians), and nonmusicians from China (hereafter termed Chinese nonmusicians). We hypothesized that there would be significant perceptual differences between Chinese and Western listeners especially for Chinese music played by Chinese instruments due to an in-group advantage. The second hypothesis was that musicians would perceive the intended affect more accurately with respect to the intended emotion than would nonmusicians.

*3. Do preference and familiarity influence affect perception?*

Previous studies have shown that familiar music induces increased pleasantness and low tension-arousal potentials (McLachlan et al., 2013; Daimi et al., 2020). Moreover, the more familiar the music, the more liked the pleasant music is. So, we hypothesized that Chinese participants would give higher valence scores and lower tension-arousal scores to Chinese music played by CIs than would Western participants (and vice versa) because of familiarity and preference.

*4. Which acoustic features are most effective for perceiving the different dimensions of affect based on the cross-cultural dataset in this study?*

Musical acoustic features mostly related to timbre and articulation were extracted to examine the relationship between timbral properties and perceived affect ratings through a linear partial least squares regression (PLSR; McAdams et al., 2017; Lembke et al., 2019).

## Materials and Methods

### Listening Experiment for Affect Ratings

#### Participants

One hundred and sixty participants took part in this listening test. Each listener group had 40 participants (Western musicians: aged 18–43years, 28 female; Western nonmusicians: aged 19–37years, 27 female; Chinese musicians: aged 18–24years, 28 female; Chinese nonmusicians: aged 18–23years, 23 female). Musicians were classified as having more than 5years of formal musical training in either the Western tradition (*M*=13.53, *SD*=6.71) or the Chinese tradition (*M*=9.62, *SD*=3.43; Zhang et al., 2020). Nonmusicians were classified as having less than 1year of formal musical training (Western: *M*=0.18, *SD*=0.42; Chinese: *M*=0.28, *SD*=0.42). Western musicians had significantly more years of formal training than Chinese musicians, *t* (78)=2.51, *p*=0.016. There was no significant difference in years of formal training between Western and Chinese nonmusicians, *t* (78)=0.91, *p*=0.37. Chinese participants were recruited in Beijing and were university students who were raised in China. All Chinese musicians had professionally studied sight singing and ear training for Western tonal music. All Chinese nonmusicians had listened to different types of Western music, such as pop, rock, classical, blues, R&B, etc. Among them, 16 participants had listened to Western classical music in the concert hall, and 28 participants had passively listened to Western classical music while doing other things. Western participants who were raised in Canada were recruited in Montreal through the student community of McGill University. None of Western participants listed Chinese music as the top three favorite music genres in either active or passive listening situations. All participants met the required hearing threshold of 20dB HL by a pure-tone audiometric test with octave-spaced frequencies from 125 to 8kHz (Martin and Champlin, 2000; ISO 398-8, 2004). Participants signed an informed-consent form and were compensated for their participation.

#### Stimuli

Four specific emotions (angry, happy, peaceful, and sad) were selected as representative examples of the affect quadrant of valence-arousal space. Two musical excerpts (one piece of Chinese and one piece of Western classical music, notations shown in Supplementary Figure S1) per emotion were chosen based on a previous study (Wang, 2018). The stimuli were recorded by having musicians on six different instruments interpret the musical excerpts with the four different affects. We recorded a total of 48 stimuli with eight musical excerpts and six different instruments.

The stimuli played by Chinese instruments were recorded in Beijing, and the stimuli played by Western instruments were recorded in Montréal. To ensure recording environment consistency, four recording studios in Beijing and three recording studios in Montréal were respectively tested for reverberation time T60 (a measure of the time required for the sound in a room to decay by 60dB), and the two recording studios with the most similar reverberation times were selected. The reverberation times T60 of the two recording studios within each octave band are shown in Table 1.

**Table 1:** The reverberation time T60 of the two recording studios.

Recording studio	125Hz	250Hz	500Hz	1kHz	2kHz	4kHz	8kHz	16kHz
Montréal T60(s)	0.330	0.323	0.343	0.290	0.318	0.325	0.295	0.235
Beijing T60(s)	0.330	0.333	0.346	0.321	0.308	0.327	0.313	0.218
Difference (%)	0.00	3.08	1.01	9.52	−3.08	0.54	5.60	−8.05

Frequencies in the first row were the center frequencies of octave bands.

A Neumann U87 microphone (Georg Neumann GmbH, Berlin, Germany) was selected for recording and placed 70cm from the performer. All stimuli were sampled at 44.1kHz with 16-bit amplitude resolution. Performers could use different techniques to express different intended affects except for tempo, which was determined by the tempo annotation on the music score of the different excerpts. All performers were from a professional conservatory and three performers were recorded for each instrument. The average duration over which Chinese performers had learned their corresponding instrument was 16.22years (*SD*=4.73), and the average duration for Canadian performers was 13.22years (*SD*=2.63). Subsequently, one Chinese and two Western musicians selected the best version of each instrument for the formal experiment. To avoid the influence of loudness on perception results, all stimuli were first calibrated based on a loudness measurement algorithm (ITU-R BS.1770-4, 2015) and then finely adjusted by ear by two volunteers.

#### Apparatus

In Canada, stimuli were stored on a Mac Pro computer (Apple Computer, Inc., Cupertino, CA, United States) and connected to Sennheiser HD650 Pro headphones for playback *via* a Grace Design m904 (Grace Digital Audio, San Diego, CA, United States) stereo monitor controller. Participants completed the experiment separately in an IAC model 120 act-3 double-wall sound-isolation booth (IAC Acoustics, Bronx, NY, United States). In China, stimuli were stored on a MacBook Pro (Apple Computer, Inc., Cupertino, CA, United States) and played back directly through Sennheiser HD650 Pro headphones. Participants completed the experiment individually in a sound-proof listening room. To ensure the consistency of sound levels on both sides, the Canadian experiment used the Brüel and Kjær Type 4153 artificial ear with the Brüel and Kjær Type 2205 sound level meter (Brüel $ Kjær, Nærum, Denmark) for sound level testing, and the Chinese experiment used the BSWA BHead230 artificial head (BSWA Technology, Beijing, China) with NTi XL2 sound level meter (NTi Audio, Schaan, Liechtenstein). The sound level was about 71dB SPL (A weighting). The experimental session was programmed with the PsiExp computer environment (Smith, 1995).

#### Procedure

**Figure 1:**
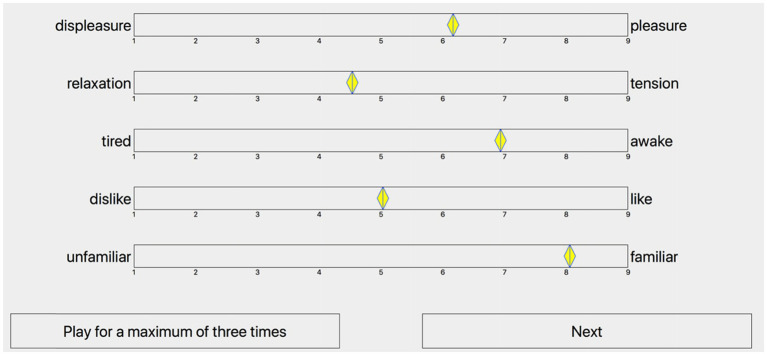
The interface for the listening test.

The experimental procedure was explained to the participants who completed four practice trials prior to the formal experiment to become familiar with the interface operation. Participants reported ratings of valence, tension arousal, energy arousal, preference, and familiarity. The interface consisted of five clearly labeled nine-point analogical-categorical scales (Weber, 1991) for each trial, as shown in Figure 1. The first three ratings measured perceived affect through a three-dimensional affect model: valence (scale endpoints labeled displeasure-pleasure), tension arousal (relaxation-tension), and energy arousal (tired-awake). Participants were instructed to judge the affect that the music was trying to express, rather than the affect that they were experiencing. The last two ratings measured preference (dislike-like) and familiarity (unfamiliar-familiar) to examine their influence on perceived affect ratings. Participants could listen to each trial a maximum of three times to reduce the impact of the familiarity rating and were also reminded that a rating of 5 equated to a neutral rating. Since there were only eight different musical excerpts in this experiment, familiarity was rated only when each musical excerpt was presented the first time. All 48 stimuli were pseudo-randomized, such that the same excerpt played on different instruments was not presented in successive trials. When participants completed all the ratings for each trial, they could click the “next” button to listen to the next trial. To measure retest reliability, participants were requested to repeat the experiment after a half an hour break. During the break time, they filled out the demographic questionnaire.

### Analysis of Acoustic Features

Several spectral, temporal, and spectrotemporal acoustical features of timbre (McAdams et al., 1995) were extracted from the 48 experiment stimuli to explore their influence on perceived affect ratings, as well as other potential features describing the articulation caused by different performing techniques. All these features are shown in Table 2 (Alías et al., 2016; Sharma et al., 2019). MIRToolbox (Lartillot and Toiviainen, 2007) and Timbre Toolbox (Peeters et al., 2011) were used to extract acoustic features. Timbre Toolbox performed accurately on individual music notes (Kazazis et al., 2017), but some algorithms especially for temporal features did not fit the melody unless individual notes were extracted first. Therefore, most of the acoustic features were calculated through MIRToolbox, except frequency modulation, amplitude modulation, and the frame energy computed on the equivalent rectangular bandwidth (ERB) input representation. The ERB was proposed by Moore and Glasberg (1983) for modeling auditory filters based on the response of the basilar membrane.

**Table 2:** Acoustic features related with timbre and performing technique.

Category	Abbreviation	Acoustic feature	Definition	Description	Values
Spectral	SpecCent	Spectral centroid	Geometric center of the spectrum	Describe shape of spectral envelope, related to the brightness and fullness perception	Mean
	SpecBrig	Spectral brightness	The amount of energy above the cut-off frequency		Mean
	SpecSpread	Spectral spread	SD of the spectrum around its mean value		Mean
	SpecSkew	Spectral skewness	Asymmetry of the spectrum around its mean value		Mean
	SpecKurt	Spectral kurtosis	Flatness of the spectrum around its mean value		Mean
	SpecFlat	Spectral flatness	The ratio between the geometric and the arithmetic mean of the energy spectral value	Estimate noisiness of the sound	Mean
	SpecEntr	Spectral entropy	Shannon entropy of the spectrum		Mean
Spectrotemporal	SpecFlux	Spectral flux	The distance between the spectrum of successive frames	Describe the degree of variation in a spectrum over time	Mean
Temporal	ZcrRate	Zero Crossing Rate	The number of times that the signal value crosses zero	Indicator of noisiness, also related to brightness perception	Mean
	AttTime	Attack time	The duration between the time of start to the end of the attack part	Describe shape of temporal envelope, related to impulsive or sustained characteristics of the sound	Mean^*^
	DecTime	Decay time	The duration of the decrease part		Mean^*^
	EffeDur	Effective duration	The time the energy envelop is above a given threshold	Estimate staccatos or legatos	Mean^*^
	EventDen	Event density	The number of notes detected per second	Estimate tempo and articulation	Mean^*^, SD^*^
	FreMod	Frequency modulation	Frequency of energy modulation	Estimate vibratos or tremolos	Mean^*^
	AmpMod	Amplitude modulation	Amplitude depth of energy modulation		Mean^*^
Dynamic	FEngERB	Frame energy of ERB	Frame energy of ERB through gammatone filter	Estimate sound energy, related to loudness	Median, IQR

* Onset first calculated to separate each tone, then acoustical features extracted to obtain statistical value.

The power spectrum estimation was applied to calculate the spectrum, which used the short-time Fourier transform (STFT) length of 8,192 sample points, with a Hann-windowed analysis of 50ms, and an overlap of 50% between successive frames (Lartillot, 2019). The final calculated spectrum was the linear magnitude spectrum. All spectral and spectrotemporal features were extracted based on the spectrum and a time series for each feature (Lartillot and Toiviainen, 2007). The mean value was calculated to represent each feature, which was the default statistical method of MIRToolbox.

A temporal envelope was needed for temporal features, which was calculated by Hilbert transform and filtered using an auto-regressive filter of infinite impulse response (Lartillot, 2019). The onset of each note was estimated in order to separate notes based on the temporal envelope, then attack time, decay time, and the effective duration of each note were extracted. The mean value of these features was taken to represent the central tendency. Event density was also extracted according to the onset of each note, then mean and SD values were computed.

The frame energy of the ERB model output was chosen to represent the dynamic of the sound. Spectra were partitioned to correspond to the human auditory system’s frequency resolution using an ERB filter and the energy of the spectrum was calculated. A bank of gammatone filters was one method of implementing ERB filters in the Timbre Toolbox (Peeters et al., 2011). For the frame energy, the STFT input representation was adopted with a length of 8,192 sample points, and a Hann window of 23.2ms with 25% overlap. The median and interquartile range were calculated as a default statistical method for time-varying features using the Timbre Toolbox (Peeters et al., 2011).

The frequency and amplitude modulation were computed for the sustaining part of the ADSR model of a musical note in Timbre Toolbox (Zhang and Bocko, 2015); therefore, the onset of each note had to be obtained first which was implemented using the MIRToolbox. The results of all the notes were averaged to represent the central tendency.

## Results

First, test-retest and Cronbach alpha reliability tests were conducted to check the validity of the results. Test-retest results on the two sets of data recorded for each participant showed that participants had good consistency for all scales, with a Pearson’s correlation *r*(7678)=0.81, *p*<0.001 for displeasure/pleasure, *r*(7678)=0.61, *p*<0.001 for relaxation/tension, *r*(7678)=0.78, *p*<0.001 for tired/awake, *r*(7678)=0.67, *p*<0.001 for dislike/like. Repeated test results for each participant were averaged to calculate the Cronbach alpha based on standardized items (mean of internal consistency) and intraclass correlation coefficients (ICC) as measures of reliability (Koo and Li, 2015). These measures indicated that all scales had very good internal consistency over the 160 participants: Cronbach alphas were 0.996 for displeasure/pleasure, 0.983 for relaxation/tension, 0.998 for tired/awake, 0.959 for dislike/like, 0.894 for unfamiliar/familiar. For these same scales, ICCs of a two-way mixed-effects model on average measures using an absolute agreement definition gave similar results: 0.995, 0.976, 0.997, 0.925, and 0.838, respectively. We will first present the listening results and then the PLSR analysis with timbre-related acoustic features.

### Listening Results

The listening test was a 4×2×4×2×3 mixed-measures design with one between-subjects factor and four repeated measures. The between-subjects factor had four LG: Western nonmusicians, Western musicians, Chinese nonmusicians, and Chinese musicians. The repeated-measures factors included melodies from two music cultures (MC: Western and Chinese), four intended musical emotions of the melodies (ME: angry, happy, peaceful, and sad), two instrument cultures (ICU: Western and Chinese) and three instrument categories (ICA: bowed chordophones, plucked chordophones, and aerophones).

There were 192 groups of variables; therefore, this experiment was not suitable for examining whether the data for each group was normally distributed. Howell (2012) has mentioned that if the largest variance is no more than four times the smallest with an equal sample size, an ANOVA is most likely to be valid. For this experiment, these ratios for valence, tension arousal, energy arousal, preference, and familiarity were 3.78, 2.12, 2.99, 3.96, and 1.88, respectively. Therefore, a mixed five-way ANOVA was conducted with two between-subjects factors (LG, MC) and three within-subject factors (ME, ICA, and ICU). To conduct analyses of the effects of within-subject factors, the Greenhouse-Geisser (*ε*<0.75) or Huynh-Feldt (*ε*≥0.75) epsilon was applied to control for the inflation of the *F* statistic due to sphericity violations. Bonferroni-corrected *post hoc* pairwise comparisons were performed for further comparison. Partial eta squared ($\eta_{p}^{2}$) was used to estimate effect size (Cohen, 1973). For all statistical tests, two-sided *p* values were used and alpha was set to 0.05. Supplementary Table S1 shows descriptive statistics (mean and SD) of each condition for all participant ratings. The full ANOVA results are presented in Supplementary Table S2.

#### The Influence of Timbre on Perceived Affect Ratings

The five-way interaction was statistically significant for all three perceived affect scales, although the effect sizes were quite small ($\eta_{p}^{2}$ =0.039, 0.031, 0.031, for energy arousal, tension arousal, and valence, respectively). Separate four-way ANOVAs for each intended emotion on each scale were conducted. Adjusting for multiple analyses for each scale, only the four-way interaction for sad on the energy scale was significant (see Supplementary Table S3).

Given that our research was primarily interested in interactions among instrument category, instrument culture, and listener group for the perceived affect ratings, this section will focus on three-way interactions involving these factors. The main effect of instrument category and instrument culture and their interaction were significant for all scales.

**Figure 2:**
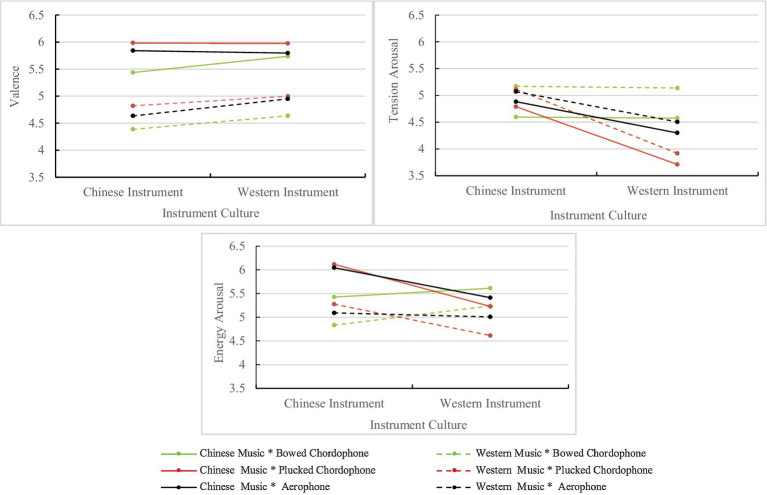
Results of the three-way interaction between instrument category, instrument culture, and musical culture of melodies for valence (upper left), tension arousal (upper right), and energy arousal (lower center) scales.

The three-way interaction effects between instrument category, instrument culture, and musical culture were significant for valence and energy arousal, but not for tension arousal, as shown in Figure 2. In each panel in Figure 2, Chinese instruments are on the left and Western instruments on the right. Data points show means for the different categories of instruments in each culture, playing either Chinese (solid line) or Western (dashed line) melodies. For valence ratings, a two-way simple effect analysis for the melodies of each musical culture indicated that there was a significant interaction between instrument category and instrument culture for Chinese music [*F*(1.94, 301.95)=17.06, *ε*=0.97, *p*<0.001, $\eta_{p}^{2}$ =0.05] but not for Western music [*F*(1.85, 288.12)=2.24, *ε*=0.92, *p*=0.11]. For Chinese music, *post hoc* comparisons revealed that the difference in mean ratings was only significant for the violin and *erhu* (*Z*=0.30, *p*<0.001). For Western music, the mean valence ratings of Western instruments were all higher than Chinese instruments for the same instrument category. Globally, plucked chordophones and aerophones show the greatest difference between Chinese and Western instruments.

A two-way simple effect analysis for musical culture on energy arousal ratings indicated that there were significant interaction effects between instrument category and instrument culture for both Chinese music [*F*(1.90, 296.59)=127.24, *ε*=0.95, *p*<0.001, $\eta_{p}^{2}$ =0.45], and Western music [*F*(1.86, 289.92)=2.24, *ε*=0.93, *p*<0.001, $\eta_{p}^{2}$ =0.43]. For Chinese music, *post hoc* comparisons revealed that the differences in mean ratings were significant for all instrument categories: violin and *erhu* (*Z*=0.18, *p*<0.001); *pipa* and guitar (*Z*=0.89, *p*<0.001); *dizi* and flute (*Z*=0.64, *p*<0.001). For Western music, *post hoc* pairwise comparisons revealed significant differences in mean ratings for the violin and *erhu* (*Z*=0.41, *p*<0.001), and for the *pipa* and guitar (*Z*=0.66, *p*<0.001), but not for the *dizi* and flute. Regardless of whether Chinese or Western music was involved, guitar ratings for tension arousal were the lowest, and flute ratings were next lowest. The other four instruments’ ratings were similar and higher than guitar and flute in tension arousal. For Chinese music, the *pipa* and *dizi* ratings were significantly higher than the other four instruments, whereas, for Western music, these two instruments were not significantly different from the other instruments.

**Figure 3:**
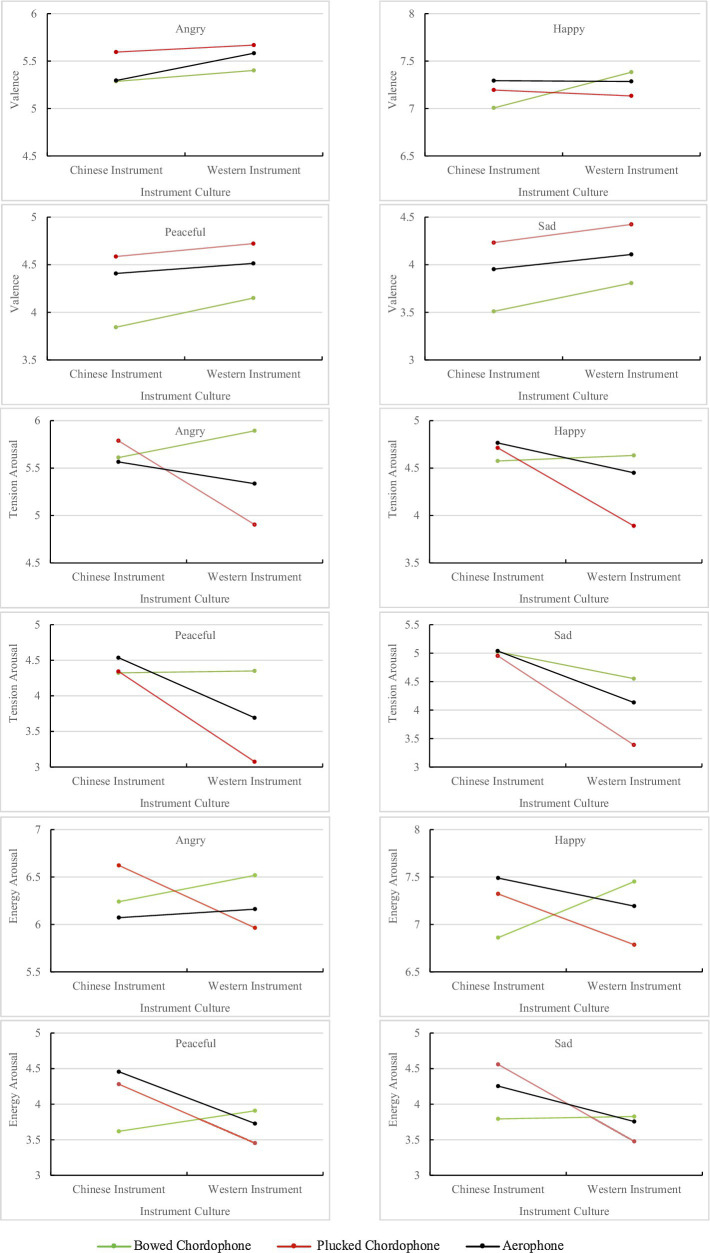
Plots of the three-way interactions between instrument category, instrument culture, and musical emotion for the three perceived affect scales.

The results of the three-way interaction effects between instrument category, instrument culture, and intended musical emotion on the mean ratings of perceived affect are displayed in Figure 3. For valence ratings, a simple effect analysis for each emotion indicated that there was a significant interaction effect between instrument category and instrument culture for angry music [*F*(1.96, 305.78)=4.54, *ε*=0.98, *p*=0.012, $\eta_{p}^{2}$ =0.03] and happy music [*F*(2, 312)=24.45, *p*<0.001, $\eta_{p}^{2}$ =0.14], but not for peaceful music [*F*(2, 312)=2.69, *p*=0.069] or sad music [*F*(2, 312)=1.22, *p*=0.27]. For angry music, *post hoc* comparisons revealed that the difference in mean ratings was significant for the violin and *erhu* (*Z*=0.12, *p*=0.039), and for the flute and *dizi* (*Z*=0.29, *p*<0.001), but not for the *pipa* and guitar. *Post hoc* comparisons for happy music revealed that the difference in mean ratings was only statistically significant for the violin and *erhu* (*Z*=0.38, *p*<0.001). For peaceful and sad music, the mean ratings of Western instruments were all higher than Chinese instruments from the same instrument category. The *erhu* was rated as having the lowest (most negative) valence of all the instruments overall.

For tension arousal ratings, a simple effect analysis indicated that there were significant interaction effects between instrument category and instrument culture on all intended emotions: angry [*F*(2, 312)=72.39, *p*<0.001, $\eta_{p}^{2}$ =0.32]; happy [*F*(2, 312)=39.94, *p*<0.001, $\eta_{p}^{2}$ =0.20]; peaceful [*F*(1.97, 207.64)=68.32, *ε*=0.99, *p*<0.001, $\eta_{p}^{2}$ =0.30]; and sad [*F*(2, 312)=46.08, *p*<0.001, $\eta_{p}^{2}$ =0.23]. For all intended emotions, the guitar ratings were the lowest and the flute ratings were next lowest. The rating differences between Chinese instruments were relatively small, whereas the differences between Western instruments were significantly larger. The order of Western instruments from high to low tension was violin, flute, and guitar for all musical emotions. Western instruments had the largest impact on tension arousal ratings.

A simple effect analysis of energy arousal indicated that there were significant interaction effects between instrument category and instrument culture for all emotion categories: angry [*F*(1.91, 298.39)=58.27, *ε*=0.96, *p*<0.001, $\eta_{p}^{2}$ =0.32]; happy [*F*(2, 312)=131.95, *p*<0.001, $\eta_{p}^{2}$=0.46]; peaceful [*F*(1.97, 207.64)=88.92, *p*<0.001, $\eta_{p}^{2}$ =0.36]; and sad [*F*(2, 312)=55.09, *p*<0.001, $\eta_{p}^{2}$ =0.26]. The guitar ratings were the lowest for all musical emotions, whereas *pipa* and *dizi* ratings were relatively high. For Western instruments, the effect on energy arousal ratings from high to low was violin, flute, and then guitar.

#### Differences in Perceived Ratings Between Listener Groups

We examined the main effect of listener group and its three-way interactions with instrument category, instrument culture, musical emotion, and musical culture for all three perceived affect ratings, as well as preference and familiarity (see Supplementary Table S2). The main effect of listener group was significant for all ratings except energy arousal.

**Figure 4:**
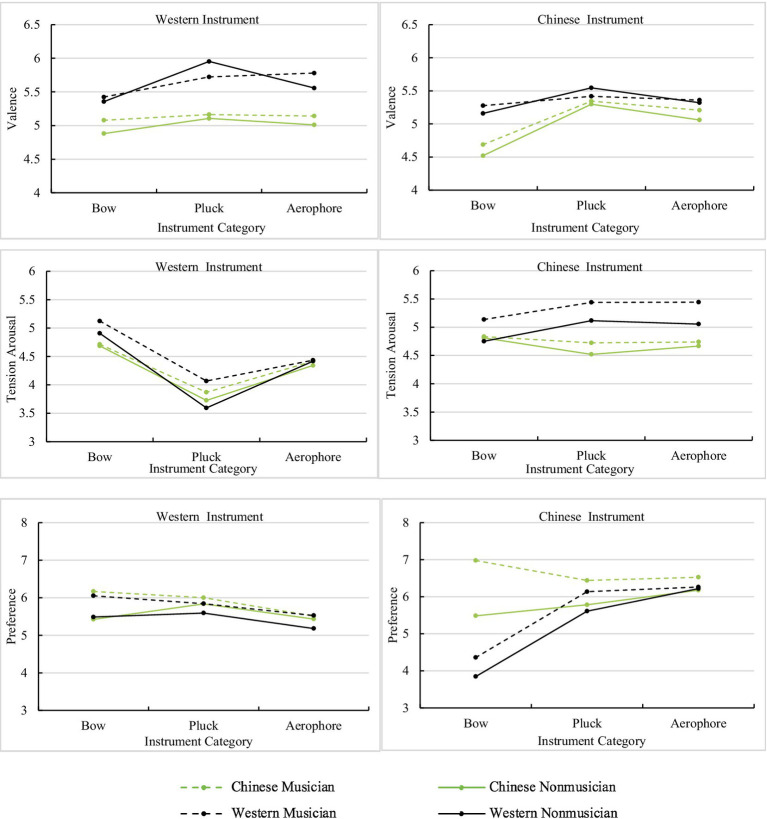
The results of the three-way interaction effect between instrument category, instrument culture, and listener group on valence, tension arousal, and preference.

The three-way interactions between listener group, instrument category, and instrument culture were significant for ratings of valence, tension arousal, and preference (Figure 4), but not for energy arousal and familiarity. For valence ratings, a simple effect analysis combining instrument category and instrument culture into a single factor of instrument indicated that the difference in mean ratings between listener groups was significant for the violin [*F*(3, 156)=5.44, *p*=0.001, $\eta_{p}^{2}$ =0.09], guitar [*F*(3, 156)=8.9, *p*<0.001, $\eta_{p}^{2}$ =0.15], flute [*F*(3, 156)=10.06, *p*<0.001, $\eta_{p}^{2}$ =0.17], and *erhu* [*F*(3, 156)=9.71, *p*<0.001, $\eta_{p}^{2}$ =0.16], but not for the *pipa* [*F*(3, 156)=0.99, *p*=0.40], and *dizi* [*F*(3, 156)=1.71, *p*=0.17]. Significant differences between listener groups depended on the musical instrument. For the guitar and *erhu*, the mean ratings of Western listeners were significantly higher than those of Chinese listeners. For the violin, *post hoc* comparisons revealed that the mean ratings of Western listeners were significantly higher than those of Chinese nonmusicians. These results indicated that participants’ cultural backgrounds had a great impact on the perceived valence rating.

For tension arousal ratings, a simple effect analysis indicated that the difference in mean ratings among listener groups was significant for violin [*F*(3, 156)=3.37, *p*=0.02, $\eta_{p}^{2}$ =0.06], guitar [*F*(3, 156)=2.99, *p*=0.033, $\eta_{p}^{2}$ =0.05], *pipa* [*F*(3, 156)=11.45, *p*<0.001, $\eta_{p}^{2}$ =0.18], and *dizi* [*F*(3, 156)=8.04, *p*<0.001, $\eta_{p}^{2}$ =0.13], but not for *erhu* [*F*(3, 156)=0.99, *p*=0.40] or flute [*F*(3, 156)=1.71, *p*=0.17]. For the violin, *post hoc* comparisons revealed that the mean ratings were significantly higher for Western musicians than for Chinese nonmusicians. For the *pipa*, the mean ratings were significantly higher for Western listeners than for Chinese nonmusicians, and the ratings of Western musicians were significantly higher than those of Chinese musicians. For the *dizi*, Western musicians rated tension arousal significantly higher than did Chinese listeners. For the guitar, the mean ratings of Western musicians were significantly higher than for Western nonmusicians. We observed that participants’ cultural backgrounds had a greater influence on the perceived tension arousal ratings than their musical backgrounds.

For preference ratings, a simple effect analysis indicated that the difference in mean ratings between listener groups was significant for the violin [*F*(3, 156)=4.42, *p*=0.005, $\eta_{p}^{2}$ =0.08], the *erhu* [*F*(3, 156)=40.23, *p*<0.001, $\eta_{p}^{2}$ =0.44], and the *pipa* [*F*(3, 156)=4.4, *p*=0.005, $\eta_{p}^{2}$ =0.08], but not for the guitar [*F*(3, 156)=0.80, *p*=0.50], the flute [*F*(3, 156)=0.73, *p*=0.54] or the *dizi* [*F*(3, 156)=0.87, *p*=0.46]. For the violin, the mean ratings of musicians were higher than those of nonmusicians. For the *erhu*, Chinese musicians’ ratings were the highest, and the mean ratings were also higher for Chinese nonmusicians than for Western listeners. For the *pipa*, the mean ratings of Chinese musicians were significantly higher than those of Western nonmusicians. We observed that participants’ cultural and musical backgrounds both had an impact on preference, and this impact was more marked for excerpts played on Chinese instruments.

**Figure 5:**
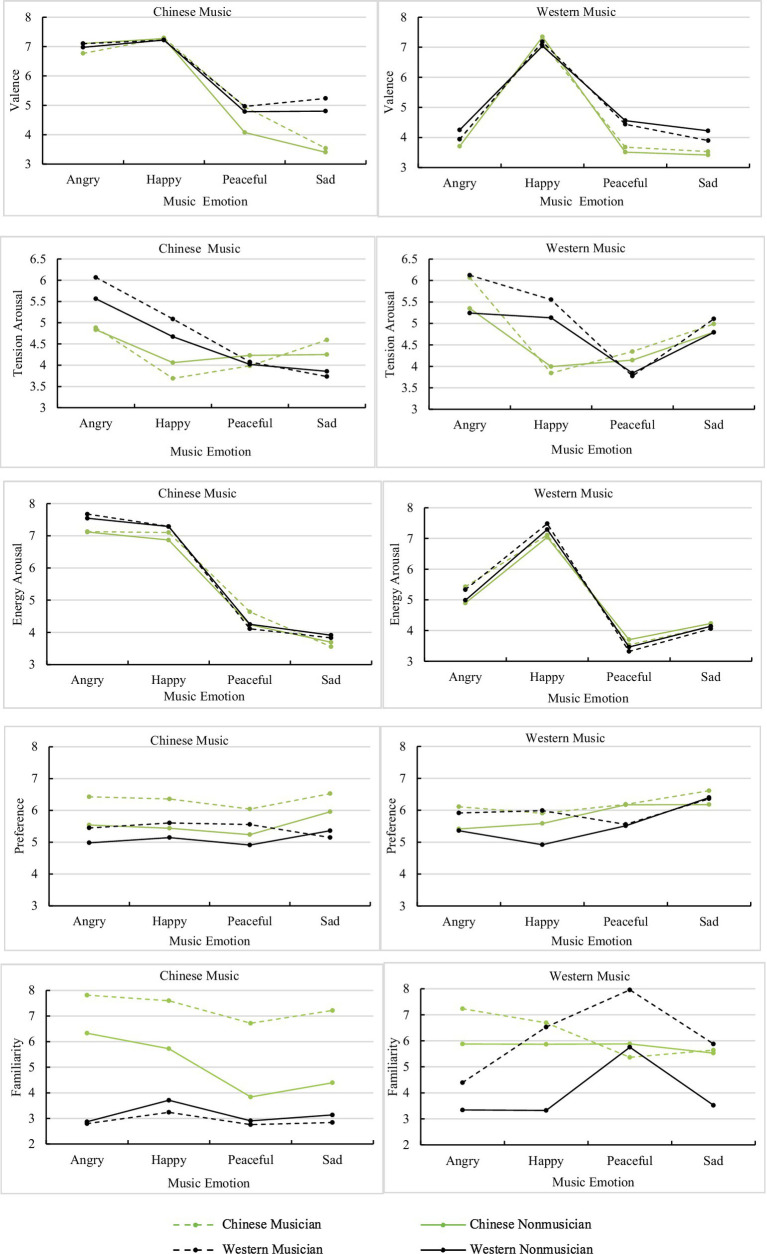
Plots of the three-way interaction effects between musical culture, musical emotion, and listener group for valence, tension arousal, energy arousal, preference, and familiarity.

The three-way interactions between listener group, musical culture, and musical emotion on different perception ratings are displayed in Figure 5. For valence ratings, a simple effect analysis by intended emotion and musical culture indicated that the difference in mean ratings between listener groups was significant for peaceful music [Western: *F*(3, 156)=6.27, *p*<0.001, $\eta_{p}^{2}$ =0.04; Chinese: *F*(3, 156)=5.28, *p*=0.002, $\eta_{p}^{2}$ =0.03] and sad music [Western: *F*(3, 156)=27.71, *p*<0.001, $\eta_{p}^{2}$ =0.15; Chinese: *F*(3, 156)=3.67, *p=* 0.014, $\eta_{p}^{2}$ =0.03], but not for angry music [Western: *F*(3, 156)=1.45, *p*=0.23; Chinese: *F*(3, 156)=0.93, *p*=0.43] or happy music [Western: *F*(3, 156)=0.73, *p*=0.54; Chinese: *F*(3, 156)=0.08, *p*=0.97]. The mean ratings of Western listeners were significantly higher than those of Chinese listeners when listening to Chinese sad music and Western peaceful music. The Western nonmusicians’ ratings were significantly higher than those of Chinese nonmusicians when listening to Western sad music. When listening to Chinese peaceful music, Chinese nonmusicians’ ratings were much lower than those of the other listener groups.

For tension arousal ratings, a similar simple effect analysis indicated that the difference in mean ratings between listener groups was significant for angry music [Western: *F*(3, 156)=10, *p*<0.001, $\eta_{p}^{2}$ =0.16; Chinese: *F*(3, 156)=8.17, *p*<0.001, $\eta_{p}^{2}$ =0.14], happy music [Western: *F*(3, 156)=19.15, *p*<0.001, $\eta_{p}^{2}$ =0.27; Chinese: *F*(3, 156)=12.09, *p*<0.001, $\eta_{p}^{2}$ =0.19], and Chinese sad music [*F*(3, 156)=5.07, *p=* 0.002, $\eta_{p}^{2}$ =0.09], but not for peaceful music [Western: *F*(3, 156)=1.82, *p*=0.15; Chinese: *F*(3, 156)=0.49, *p*=0.69] and Western sad music [*F*(3, 156)=0.75, *p*=0.52]. For Chinese angry music and Chinese and Western happy music, the mean ratings of Western listeners were significantly higher than those of Chinese listeners. For Chinese sad music, to the contrary, the ratings were higher for Chinese listeners than for Western musicians. For Western angry music, musicians’ ratings were significantly higher than those of nonmusicians. A rating of 5 indicated a neutral score, so musicians’ ratings of Western angry music were more clearly differentiated than those of nonmusicians. Therefore, both the musical culture of the melodies and musical training affect tension arousal ratings.

For energy arousal ratings, a simple effect analysis indicated that the differences in mean ratings between listener groups were significant for Chinese peaceful music [*F*(3, 156)=2.7, *p*=0.047, $\eta_{p}^{2}$ =0.02], and all angry music [Chinese: *F*(3, 156)=5.24, *p*=0.002, $\eta_{p}^{2}$ =0.09; Western: *F*(3, 156)=2.37, *p*=0.047] and happy music [Chinese: *F*(3, 156)=3.28, *p*=0.023, $\eta_{p}^{2}$ =0.06; Western: *F*(3, 156)=2.82, *p*=0.041, $\eta_{p}^{2}$ =0.05], but not for Chinese sad music [*F*(3, 156)=1.32, *p*=0.27] and the other Western music [peaceful: *F*(3, 156)=0.92, *p*=0.43; sad: *F*(3, 156)=0.17, *p*=0.92]. For Chinese angry music, Western listeners’ mean ratings were significantly higher than for Chinese listeners. For Chinese and Western happy music, Western listeners’ mean ratings were significantly higher than Chinese nonmusicians. For Chinese peaceful music, Chinese musicians’ mean ratings were higher than Western musicians. From the results, we observed that participants’ cultural backgrounds had a greater impact on energy arousal ratings than their musical backgrounds.

For ratings of preference, a simple effect analysis indicated that the interaction effect between listener groups and musical emotions was significant both for Chinese music [*F*(9, 468)=3.88, *p*<0.001, $\eta_{p}^{2}$ =0.07], and Western music [*F*(9, 468)=4.22, *p*<0.001, $\eta_{p}^{2}$ =0.08]. The Chinese musicians’ mean ratings were much higher than the other three groups for all Chinese music. For Western angry music, musicians’ mean ratings were higher than nonmusicians. For Western happy music, musicians’ mean ratings were significantly higher than Western nonmusicians.

For ratings of familiarity, a simple effect analysis indicated that the interaction effect between listener groups and musical emotions was significant both for Chinese music [*F*(9, 468)=5.48, *p*<0.001, $\eta_{p}^{2}$ =0.10], and Western music [*F*(9, 468)=8.14, *p*<0.001, $\eta_{p}^{2}$ =0.14]. Chinese musicians’ mean ratings were significantly higher than the other three groups for Chinese music, which indicated that they were very familiar with all Chinese music. Chinese nonmusicians were familiar with Chinese angry and happy music. Western listeners were not familiar with Chinese music at all. Western musicians were familiar with most Western music except Western angry music. Chinese listeners were also familiar with Western music, and this result was consistent with the questionnaire results of the participants. Western nonmusicians were not familiar with most Western music except Western peaceful music.

**Figure 6:**
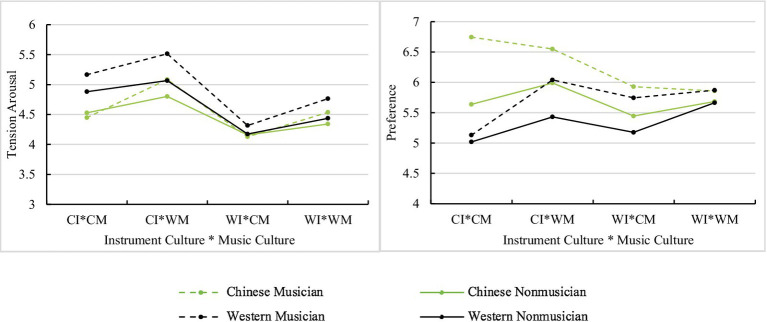
The results of the three-way interaction effects between listener group, instrument culture, and musical culture on tension arousal and preference. CI, Chinese instruments; CM, Chinese music; WI, Western instruments; and WM, Western music.

The three-way interaction effects of listener group, instrument culture, and musical culture were only significant for tension arousal and preference. For tension arousal ratings (Figure 6), a simple effect analysis indicated that there were significant differences between listener groups for Chinese music played by Chinese instruments [*F*(3, 156)=8.78, *p*<0.001, $\eta_{p}^{2}$ =0.14], and Western music played by Chinese instruments [*F*(3, 156)=6.50, *p*<0.001, $\eta_{p}^{2}$ =0.13], but not for Chinese music played by Western instruments [*F*(3, 156)=0.62, *p*=0.61], or by Western music played by Western instruments [*F*(3, 156)=2.5, *p*=0.06]. The ratings of Western listeners were higher than those of Chinese listeners for Chinese music played by Chinese instruments, which demonstrated that in-group advantage influenced tension arousal. The ratings of Western musicians were higher than those of Chinese nonmusicians for Western music played by Chinese instruments, which meant that instrument culture might have more influence on tension arousal perception than musical culture.

A simple effect analysis of preference indicated that there were significant differences between listener groups for Chinese music played by Chinese instruments [*F*(3, 156)=31.54, *p*<0.001, $\eta_{p}^{2}$ =0.38], Chinese music played by Western instruments [*F*(3, 156)=9.36, *p*<0.001, $\eta_{p}^{2}$ =0.15], and Western music played by Chinese instruments [*F*(3, 156)=2.99, *p*=0.033, $\eta_{p}^{2}$ =0.05], but not for Western music played by Western instruments [*F*(3, 156)=0.41, *p*=0.74]. The effect size indicated that the differences in listener group preferences were greatest when they listened to Chinese music played by Chinese instruments, especially between Chinese musicians and Western participants.

#### Correlation Analysis of Perceived Affect Ratings, Preference, and Familiarity

The above analysis indicated that the four listener groups’ perceptions were significantly different along several factors. In order to explore the differences in the correlation between the three emotional dimensions among listener groups, and the influence of preference and familiarity on emotional perception, a Pearson correlation analysis was conducted separately for each listener group according to participants’ mean ratings of valence, tension arousal, energy arousal, preference, and familiarity for the 48 conditions, as shown in Table 3. Valence and energy arousal were strongly positively correlated for all listeners. Tension arousal was moderately negatively correlated with energy arousal for Western nonmusicians and strongly negatively correlated for Western musicians, but there was no correlation between them for Chinese listeners. Preference had more significant positive correlation with valence for nonmusicians than for musicians, which indicated that musicians distinguished between valence and preference better than nonmusicians. For Chinese listeners, familiarity was moderately positively correlated with valence and energy arousal, but very weakly positively correlated with tension arousal. For Western listeners, familiarity was very weakly to weakly correlated with all other perceived ratings. Chinese listeners were familiar with most of the stimuli; therefore, these results seemed to indicate that familiarity with the music might influence the perception of valence and energy arousal. There was a weak positive correlation between familiarity and preference for Chinese nonmusicians.

**Table 3:** Pearson’s correlation coefficients of ratings of perceived valence, tension arousal, energy arousal, preference, and familiarity.

	Western nonmusician				Western musician			
	Valence	Tension	Energy	Preference	Valence	Tension	Energy	Preference
Tension	−0.24				−0.19			
Energy	0.91^***^	−0.61^***^			0.78^***^	−0.75^***^		
Preference	0.54^***^	−0.49^***^	0.23		−0.11	−0.47^**^	−0.31^*^	
Familiarity	−0.27	0.32^*^	−0.40^**^	0.18	−0.29^*^	−0.08	−0.24	0.07
	Chinese nonmusician				Chinese musician			
Tension	−0.27				−0.49^***^			
Energy	0.96^***^	−0.01			0.91^***^	−0.11		
Preference	0.74^***^	−0.47^**^	0.68^***^		0.44^**^	−0.48^**^	0.36^*^	
Familiarity	0.51^***^	0.22	0.60^***^	0.46^**^	0.51^***^	0.08	0.58^***^	0.26

*df=46.*

* *p<0.05;*

** *p<0.01;*

*** *p<0.001*.

#### Hierarchical Cluster Analysis Between Six Instruments

**Figure 7:**
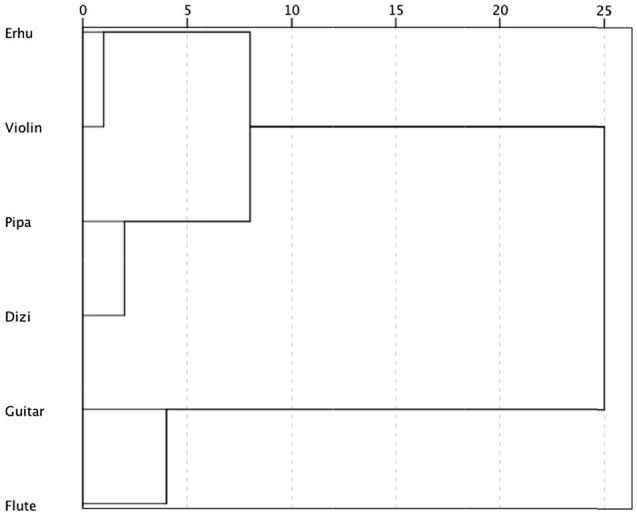
Hierarchical cluster analysis between six instruments based on mean ratings across participants of perceived valence, tension arousal, and energy arousal.

To explore the similarity of affect perception between instruments, a hierarchical cluster analysis between six instruments was conducted based on the mean ratings across participants of perceived valence, tension arousal, and energy arousal. Squared Euclidean distance was adopted as a proximity measure. According to the results (Figure 7), the *erhu* and violin were very similar and somewhat similar to the *pipa* and *dizi*, but very different from the guitar and flute. The *pipa* and *dizi* were very similar, and the guitar and flute were very similar.

### Partial Least-Squares Regression

The PLSR was performed to examine the relationship between acoustic features and perceived affect ratings. PLSR couples multiple linear regression with principal component analysis, and also allows collinearity among variables, with collinear variables being represented parsimoniously in principal components (PCs; Geladi and Kowalski, 1986).

A 6-fold cross-validation model was applied to the PLSR model by partitioning the *n* cases into six subsets. The model was trained on five subsets and the error in predicting the remaining subset was assessed. The procedure of training and prediction was repeated for all permutations of subsets. *R^2^* and *Q^2^* are generally two metrics that evaluate the performance of a PLSR model. *R^2^* evaluates the explanatory power of the model, and *Q^2^* describes the predictive power (McAdams et al., 2017; Lembke et al., 2019). To assess the relative importance of independent variables in each PLSR, each independent variable was assessed by a variable importance in projection (VIP) score, and a VIP score greater than one was generally considered a significant contribution (Chong and Jun, 2005; Janes et al., 2008). The SIMPLS algorithm (De Jong, 1993) was applied to the PLSR and implemented in MATLAB.

In our study, the PLSR independent variables were 18 acoustic features (shown in Table 2) for each of the 48 stimuli. Based on the ANOVA results, the cultural background of participants had a great influence on emotional perception, so the six dependent variables tested were the mean ratings of Western and Chinese participants separately for valence, tension arousal, and energy arousal. Two PC were considered in PLSR models of all three affect dimensions based on the *Q^2^* criterion computed by cross validation (Stone, 1974; Titin et al., 2018), which meant that the PC was significant and selected when the predicted variance *Q^2^* was larger than 0.05. The performances of each of the three affect dimensions are displayed in Table 4.

**Table 4:** *R^2^* and *Q^2^* results for partial least squares regression (PLSR) models for predicting perceived valence, tension arousal, and energy arousal, as well as component-wise contributions along the two principal components (PCs).

	Western participants				Chinese participants			
Dependent variables	*R^2^*	*Q^2^*	PC1	PC2	*R^2^*	*Q^2^*	PC1	PC2
Valence	0.48	0.32	0.22	0.38	0.55	0.44	0.32	0.31
Tension arousal	0.70	0.65	0.44	0.18	0.36	0.22	0.44	0.13
Energy arousal	0.70	0.62	0.37	0.25	0.67	0.60	0.38	0.25

#### Valence

**Figure 8:**
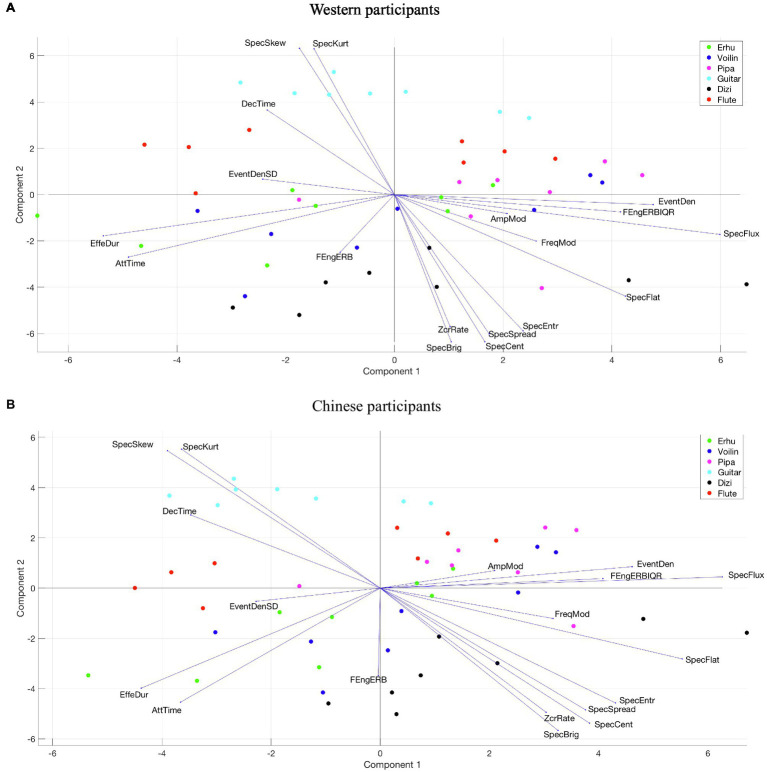
Loadings and scores across two PCs of PLSR for valence. **(A)** Western participants. **(B)** Chinese participants.

Figure 8 visualizes the PLSR loadings (vectors) and scores (circles) for valence across two PCs. Different colored circles represent stimuli played by different musical instruments. Longer vectors indicate that acoustic feature loadings contributed more strongly, and the orientations indicate the PCs by which they were primarily influenced.

The PLSR result of Western participants is very similar to that of Chinese participants, although there are differences in the percentage of explained variance for two PCs. PC1 in both groups is related to two factors: one is the spectrotemporal feature described by spectral flux; the other one includes temporal features, described by event density, effective duration, and attack time. The musical stimuli with positive coordinates in PC1 have more spectrum energy variation over time and are performed with shorter note durations with sharp attacks. Most of the stimuli played by the *pipa* have positive scores in PC1.

PC2 appears to be influenced by a collinear set of spectral features falling slightly oblique to the PC axis, such as spectral skewness, spectral kurtosis, spectral brightness, and spectral centroid. The PC2 coordinates are more negative, which means that the musical stimuli have greater high-frequency energy and wider spectral distribution. All stimuli played by the guitar and most stimuli played by the flute and the *pipa* have positive scores in PC2, whereas all stimuli played by the *dizi* and most stimuli by the *erhu* and violin have negative scores.

#### Tension Arousal

**Figure 9:**
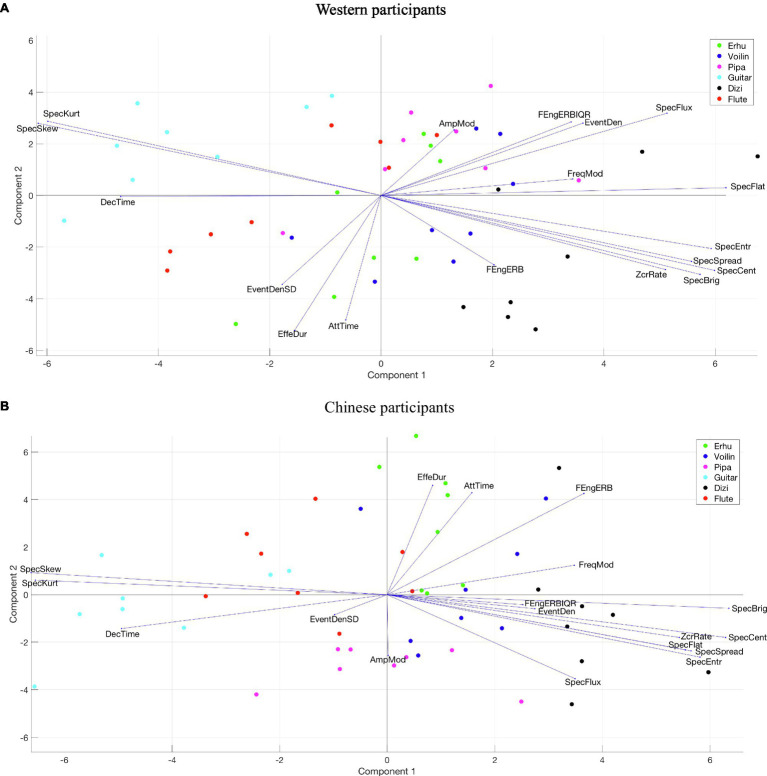
Loadings and scores across two PCs of PLSR for tension arousal. **(A)** Western participants. **(B)** Chinese participants.

The PLSR loadings and scores for tension arousal across two PCs are displayed in Figure 9. The results of Western and Chinese participants are different. For Western participants, PC1 is highly related to spectral features that quantify how noisy the sound is and describe the spectral energy distribution, such as spectral flatness, spectral entropy, spectral centroid, and spectral skewness. The more positive the PC1 coordinate, the more high-frequency energy, the wider the spectral distribution, and the more noise-like the musical stimuli are score results indicate that all music stimuli played by the *dizi* and most stimuli played by the *pipa* have positive scores on PC1, whereas all stimuli played by the guitar and most stimuli played by flute have a negative score on PC1. PC2 is influenced by temporal features falling slightly oblique to the PC axis, which includes effective duration, attack time, event density SD, and amplitude modulation. The musical stimuli with positive coordinates on PC2 were performed with a shorter note duration and vibrato articulation.

For Chinese participants, PC1 is highly related to spectral features describing the spectral energy distribution, such as spectral skewness, spectral kurtosis, spectral brightness, and spectral centroid. The more positive the PC1 coordinate, the more high-frequency energy the musical stimuli possess. PC2 is influenced by temporal features falling slightly oblique to the PC axis, which included effective duration, attack time, and frame energy of ERB.

#### Energy Arousal

**Figure 10:**
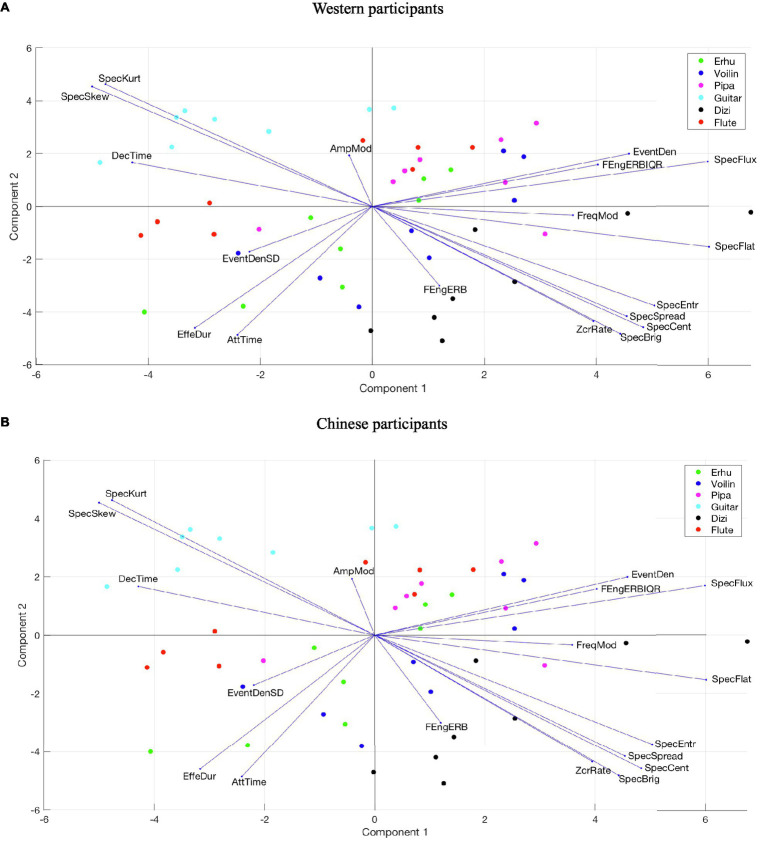
Loadings and scores across two PCs of PLSR for energy arousal. **(A)** Western participants. **(B)** Chinese participants.

Figure 10 visualizes the PLSR loadings and scores for mean energy arousal ratings across two PCs. The results of Western and Chinese participants are almost the same. PC1 is highly related to two acoustic features: spectral flatness and spectral flux. The more positive the PC1 coordinate, the more spectrum energy variation over time and noisiness characterize the music stimuli. The score results indicate that most of the musical stimuli played by the *pipa* and *dizi* have positive PC1 scores, whereas most of the stimuli played by guitar have negative PC1 scores.

PC2 is influenced by two factors: firstly temporal features, described by attack time and effective duration; secondly spectral features falling slightly oblique to the PC axis, such as spectral skewness, spectral kurtosis, spectral brightness, and spectral centroid. The musical stimuli with negative coordinates on PC2, have more high-frequency energy, wider spectral distribution, and were performed with a shorter note duration. PLSR scores indicate that all music stimuli played by the *dizi* have negative PC2 scores, while all stimuli played by guitar have positive PC2 scores.

#### Important Acoustic Features of Different Affective Dimensions

We identified the important acoustic features of different emotional dimensions with the VIP scores greater than one from the PLSR models. The top five important acoustic features for three emotional dimensions are shown in Supplementary Table S4. For valence, there is no big difference between Western and Chinese participants. Spectral flux is the most important feature. Effective duration, attack time, and event density are temporal features and all related to performance techniques. In summary, greater valence is associated with more spectral variation, more impulsive-type note envelopes (such as staccato, pizzicato) with a sharp attack, and more dynamic range. Although, we mention above that positive valence is also correlated with more high-frequency energy, it seems that high-frequency energy is not a very important factor based on our cross-cultural dataset.

Comparing the results of Chinese and Western participants, the acoustic features that affect the perception of tension arousal are quite different. For Western participants, music stimuli with higher tension arousal have more spectrum energy variation over time, wider spectral distribution, and noisier sounds with sharp decay, whereas for Chinese participants, positive tension arousal is coherent with more vibrato sounds with different note durations, greater temporal energy, wider spectrum distribution, and more high-frequency energy.

The important acoustic features for energy arousal are similar to the valence result, and there are only small differences between the two cultural groups of listeners. Spectral flux is the most important feature of energy arousal. Higher energy arousal corresponds with more spectral variation, more impulse-type note envelopes with a sharper attack, and more dynamic range.

## Discussion

The primary aim of this study was to examine three main issues: (1) how timbre impacts the perception of affect in Western and Chinese classical music with different emotional character, (2) whether participants’ cultural background, musical background, preference, and familiarity with the music influence the perception of affect based on this cross-cultural dataset, and (3) which acoustic features are the most effective factors involved in the perception of different dimensions of affect according to the PLSR results.

### The Influence of Timbre on Affect Perception

The perceived valence ratings indicate that the *erhu* had the lowest rating for any musical culture or any musical emotion. Regardless of whether the music was Chinese or Western, the plucked chordophone valence ratings were relatively high, whereas bowed chordophone scores were relatively low. Combing the scores and loadings from the PLSR model for valence, all guitar stimuli had positive scores on PC2 and most *pipa* stimuli had positive scores on PC1, which means that positive valence is associated with low-frequency spectral energy, high spectral variation, and short note duration with a sharp attack. Similar positive correlations between a low ratio of high-frequency to low-frequency energy and valence have been found in previous perceptual research (Kidd and Watson, 2003; Ilie and Thompson, 2006; Eerola et al., 2013), whereas the opposite observation was found by McAdams et al. (2017).

Independently of musical culture or musical emotion, the guitar tension arousal ratings were the lowest, and the flute scores were the second lowest, whereas all other instruments’ ratings were similar. The *dizi* had the highest scores for happy, peaceful, and sad music. According to the scores and loadings from the PLSR model for tension arousal, all guitar stimuli and most of the flute stimuli had negative scores on PC1, whereas all *dizi* stimuli had positive scores on PC1, which indicates that greater tension arousal is correlated with more high-frequency energy, wider spectral distribution, and a more noise-like spectrum. The finding on high-frequency energy corresponded with previous studies relating to the perception of emotion in music (McAdams et al., 2017) and speech (Banse and Scherer, 1996; Johnstone and Scherer, 2000).

For perceived energy arousal ratings, guitar scores were the lowest and most scores of *pipa* and *dizi* were relatively high, independently of musical culture or musical emotion. Based on the scores and loadings from the PLSR model for energy arousal, all stimuli played by guitar had positive PC2 scores, whereas all stimuli played by *dizi* and most of the stimuli played by *pipa* had positive PC1 scores. These results indicate that higher energy arousal is carried by greater spectral variation, a noisier spectrum, greater high-frequency energy, and shorter note duration with a sharp attack. This result is coherent with the findings of many studies that higher energy arousal is correlated with brighter sounds (Eerola et al., 2013; McAdams et al., 2017) with sharper attacks (Eerola et al., 2013).

When combining the results between PLSR and cluster analysis, it seems that the similarity in affect perception between instruments was primarily coherent with timbre-related acoustic features caused by instrument physical characteristics or performing techniques. These acoustic features include two main aspects. One is spectral energy distribution, characterized by the spectral centroid, spectral brightness, spectral skewness, and spectral spread. The other one is the shape of the temporal envelope of musical notes, which is related to articulation such as staccato, vibrato, and legato, described by attack time, amplitude modulation, effective duration, and event density.

### Differences Between Listener Groups

In general, the cultural background of participants had a greater impact on their emotional perception than their musical background, although both play a role. The differences in perception between Chinese and Western participants on valence and tension arousal were more salient. Regardless of the instrument or the cultural origin of the music, Western participants usually had significantly higher scores than Chinese participants on these affect dimensions. For example, Western participants’ ratings of perceived valence for all instruments were higher than those of Chinese participants, which might be related to the mode of expression. Cohen and Gunz (2002) have argued that Westerners are more inclined to project egocentric emotional perspective on others, whereas Easterners are more likely to engage in relational projection. From the perspective of the current study, we argue that Westerner participants’ expression of opinions is more direct, whereas Chinese participants’ expression is more implicit. Therefore, Westerners’ valence score deviated further from the neutral score of 5. In addition, this difference was potentially correlated with familiarity. Western participants had higher tension arousal scores when listening to Chinese musical instruments, especially the *pipa* and *dizi,* than Chinese participants. This might be because Western participants were not familiar with Chinese musical instruments, which made it easier for them to evoke tension, or perhaps the unfamiliarity itself induced tension that could affect the perceptual ratings.

Consistent with the hypothesis that musicians would be more accurate in their affect perception, only one observation confirmed this idea: tension arousal scores of musicians were significantly higher than those of nonmusicians when listening to Western angry music. Musicians’ scores deviated more from the neutral score of 5 than nonmusicians, which might indicate that due to years of music training and enculturation, musicians were more confident in making judgments.

Regarding the influence of the in-group advantage on affect perception, Chinese and Western participants had greater differences in perceived tension arousal ratings when listening to Chinese musical instruments playing Chinese music than in other cases. This phenomenon was more obvious with ratings of preference. Due to the high recognition of Chinese instruments and music from the same culture, Chinese participants’ preference for Chinese music played by Chinese instruments was significantly higher than that of Western participants.

Participants’ preference and familiarity did impact affect perception, and there were significant differences between different participants. Preference was correlated with valence moderately in Western nonmusicians and strongly in Chinese nonmusicians, but weakly to very weakly for musicians, which indicates that nonmusicians are more likely to confuse the perceived measure of valence and the felt measure of preference. The impact of familiarity on valence and energy arousal in Chinese participants is more salient because Western music is ubiquitous in China, which means that Chinese participants are familiar with the style of most stimuli. Accordingly, we can infer that familiarity potentially plays a role in valence and energy arousal ratings when participants are familiar with the stimuli. However, the correlation between familiarity and tension arousal was very weak.

Familiarity was positively correlated with preference for Chinese nonmusicians. Two potential conclusions related to the effects of repetition on preference might be drawn: one is “mere exposure” (Zajonc, 2001), and the other is “inverted-U theory” (Hargreaves, 1986). Based on the research of Zajonc (2001), preference induced by the mere exposure effect depends on the objective history of exposures, instead of subjective impression of familiarity. According to “inverted-U theory,” the preference for initially unfamiliar music should be low; that it should rise to a peak with increasing exposure and familiarity; and then decline with further exposure. In our case, there is a correlation between preference and familiarity for Chinese nonmusicians, whereas there is no relationship between them for other listener groups. These findings are more consistent with the “inverted-U theory.” The order of the familiarity of experimental stimuli across different listener groups from high to low is Chinese musicians, Chinese nonmusicians, and then Western participants. Based on the “inverted-U theory,” moderate familiarity can arouse the highest preference, which is the situation of Chinese nonmusicians.

### Limitation of Performance Expression

The present study has limitation of performance expression. Only one performer was included in each condition of our study. Based on the *lens model* proposed by Juslin (2000), different performers express specific emotions by means of a number of variable cues (such as articulation, sound level). In our study, the deviation of performance expression might cause the cultural variations in musical material and instrument timbre which would influence the listener’s emotional perception. While Juslin (2000) found that professional performers were more consistent in their cue utilization to communicate particular emotions, and the variance among them was the extent to which the performer’s cue utilization matched the listener’s cue utilization. It is unclear that if these findings work in the cross-cultural context. Therefore, further experiments should be conducted using different performers to reveal more information about the influence of different performance expressions on emotion perception.

## Conclusion

This research explored the influence of timbre on perceived affect ratings using a cross-cultural approach. The explanation for the similarity of perceived affect ratings between instruments was the similar timbral acoustic features that were caused by the physical characteristics of specific instruments and performing techniques. Neither instrument category nor instrument culture was a prominent explanatory factor. Participants’ cultural backgrounds had a greater impact on affect perception than their musical backgrounds. Of course, due to years of music training, musicians had clearer judgments and exhibited more complex affect perception. In addition, musicians distinguished more clearly between a perceived measure such as valence and a felt measure such as preference. When participants were familiar with the stimuli, this potentially played a role in the perception of valence and energy arousal. According to the linear PLSR results, the important acoustic features for valence and energy arousal were similar, which related mostly to spectral variation, the shape of the temporal envelope, and the dynamic range. The important acoustic features for tension arousal described the shape of the spectral envelope, noisiness, and the shape of the temporal envelope.

Future research should further investigate whether the influence of timbre on emotional perception is different for different listener groups, and the potential factors such as performance expression and socio-cultural factors that may be involved in these differences, especially how socio-cultural factors (including language, ways of cognition) contribute to affect perception. In addition, the affect perception of music changes dynamically over time. It is important to consider which musical elements trigger such emotional changes, which will provide an effective theoretical framework for composition and orchestration.

## Data Availability Statement

The original contributions presented in the study are included in the article/Supplementary Material, further inquiries can be directed to the corresponding author.

## Ethics Statement

The studies involving human participants were reviewed and approved by the Research Ethics Boards of McGill University and the Communication University of China. The patients/participants provided their written informed consent to participate in this study.

## Author Contributions

XW, LH, and SMc designed the study. YW, XW, and LH collected the data. XW conducted acoustic analyses. XW, YW, and SMc conducted data analyses. XW and SMc drafted the manuscript. All authors edited and approved the manuscript.

## Funding

Funding was provided by the Science Foundation of Communication University of China (HG1608-1) to XW and grants from the Canadian Natural Sciences and Engineering Research Council (RGPIN 2015-05280), the Fonds de recherche du Québec-Société et culture (SE-171434), and a Canada Research Chair (950-223484) awarded to SMc.

## Conflict of Interest

The authors declare that the research was conducted in the absence of any commercial or financial relationships that could be construed as a potential conflict of interest.

## Publisher’s Note

All claims expressed in this article are solely those of the authors and do not necessarily represent those of their affiliated organizations, or those of the publisher, the editors and the reviewers. Any product that may be evaluated in this article, or claim that may be made by its manufacturer, is not guaranteed or endorsed by the publisher.
