# Supplementary material for: A Cross-Cultural Analysis of the Influence of Timbre on Affect Perception in Western Classical Music and Chinese Music Traditions
Source: Front Psychol. 2021 Sep 29;12:732865. doi: 10.3389/fpsyg.2021.732865 (PMC8511703; doi:10.3389/fpsyg.2021.732865)
Supplement: Supplementary file 1 [file Data_Sheet_1.PDF]

Supplementary Materials for

Wang, X, Wei, Y., Heng, L. & McAdams, S., A cross-cultural analysis of the influence of timbre on affect perception in Western classical music and Chinese music traditions. *Frontiers in Psychology*.

1. Figure S1. Musical notations for stimuli

Chinese Music Excerpts

Bao Wei Huang He 保卫黄河

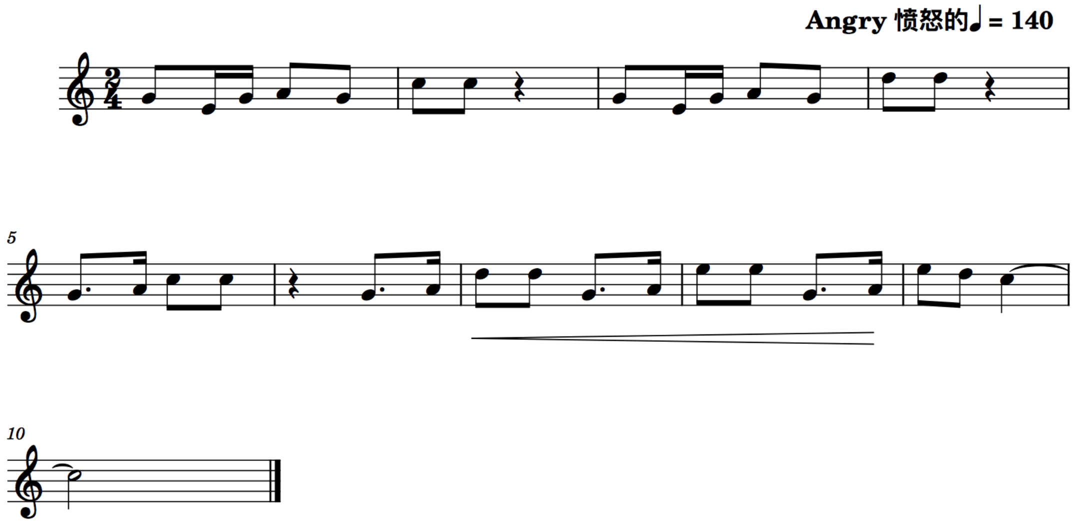

Er Quan Ying Yue 二泉映月

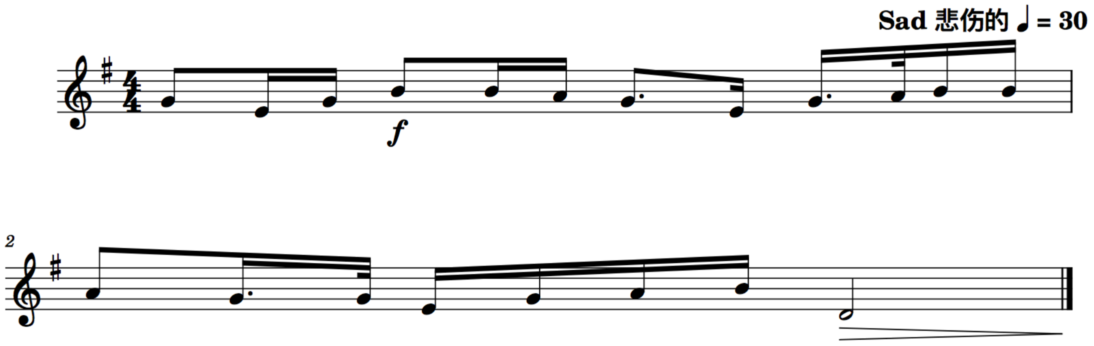

Hua Hao Yue Yuan 花好月圆

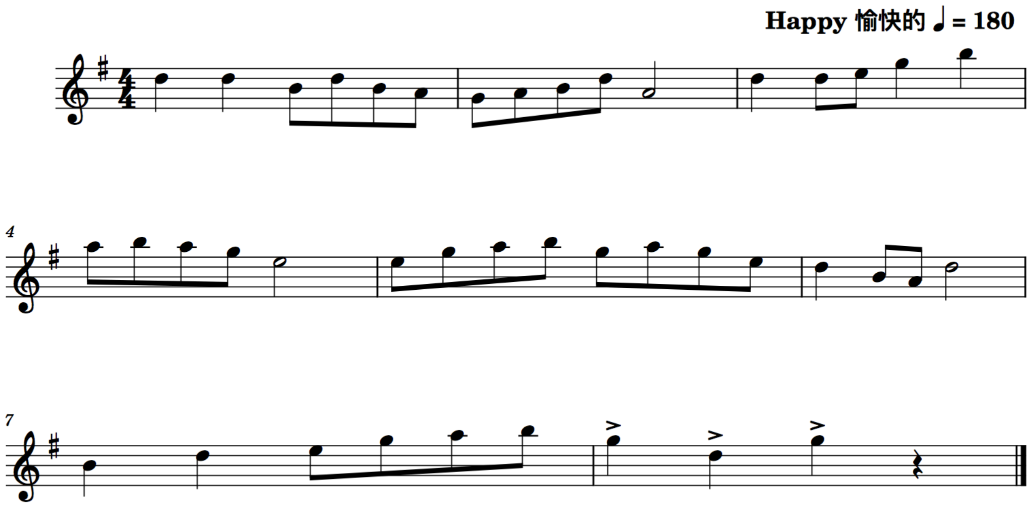

Spring River Flower Moon Night 春江花月夜

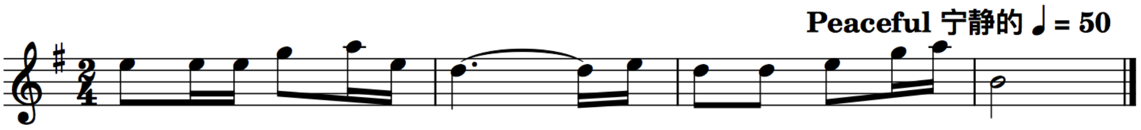

Western Music Excerpts

Zigeunerweisen 流浪者之歌

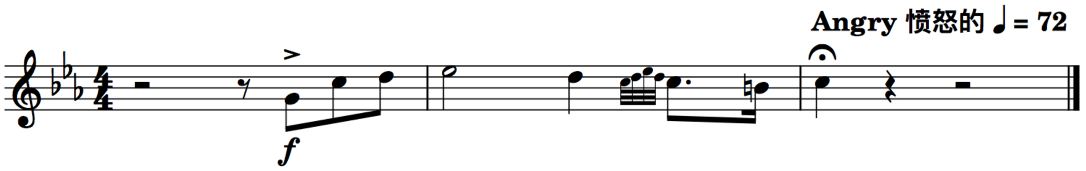

Schindler's List 辛德勒名单

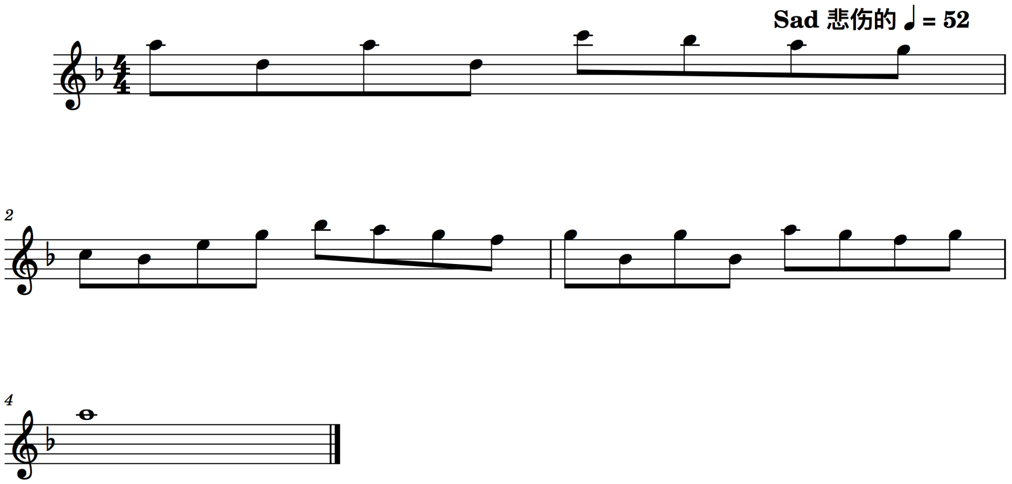

Humoresque 幽默曲

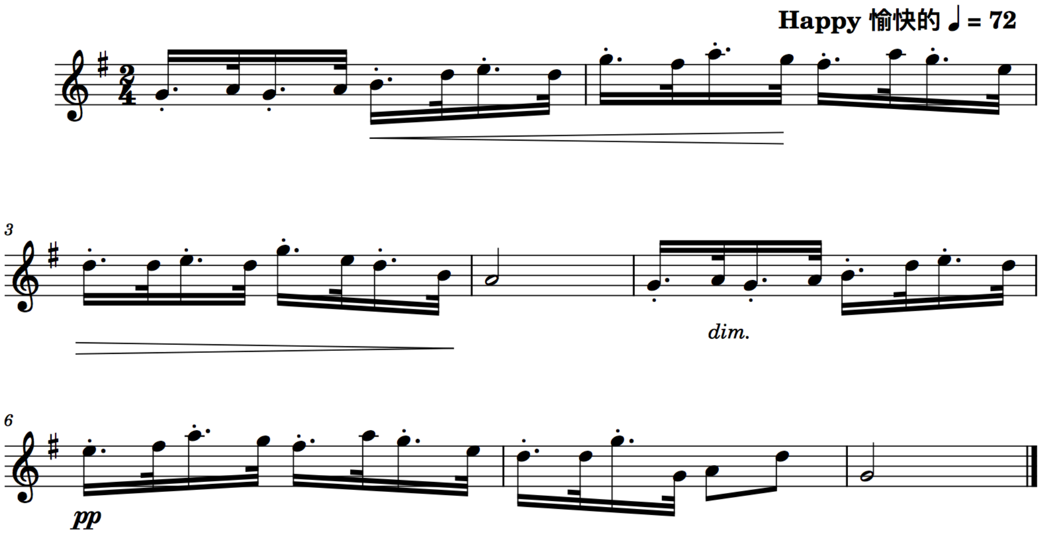

Green Sleeves 绿袖子

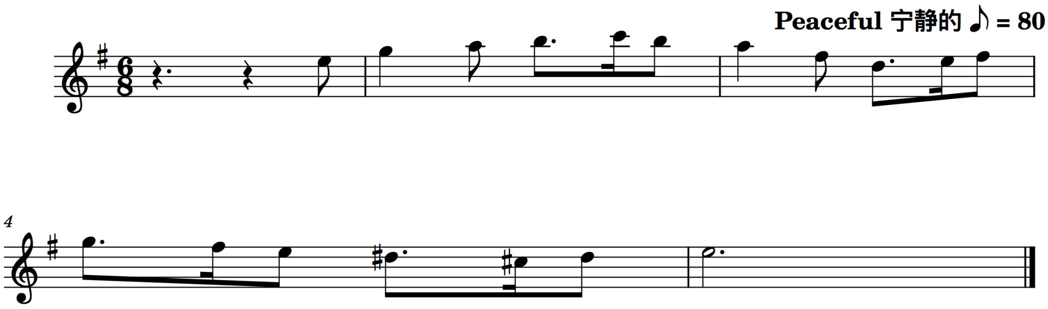

2. Tables S1. Descriptive statistics (mean (M) and standard deviation (SD)) of each condition for all participant ratings

Western Musician Results																		
Affect Dimensions	Culture		Chinese Music								Western Music							
	Affect		Angry		Happy		Peaceful		Sad		Angry		Happy		Peaceful		Sad	
	Descriptive		*M*	*SD*	*M*	*SD*	*M*	*SD*	*M*	*SD*	*M*	*SD*	*M*	*SD*	*M*	*SD*	*M*	*SD*
**Valence**	Chinese Instruments	*Erhu*	6.96	1.40	7.22	.93	4.57	1.64	5.06	1.53	3.80	1.52	6.91	1.14	4.08	1.56	3.61	1.60
		Pipa	7.12	1.25	7.05	.98	5.28	1.26	4.88	1.30	3.95	1.23	7.04	1.35	4.21	1.77	3.83	1.41
		Dizi	6.85	1.41	7.23	1.01	4.84	1.23	5.31	1.23	3.62	1.12	6.99	1.72	4.40	1.52	3.64	1.20
	Western Instruments	Violin	7.10	1.25	7.38	1.00	4.52	1.70	4.82	1.68	4.16	1.59	7.23	1.13	4.12	1.98	3.93	1.87
		Guitar	7.29	1.16	7.10	.97	5.28	1.57	5.66	1.58	4.08	1.62	7.25	1.06	4.79	1.86	4.32	2.00
		Flute	7.26	1.02	7.34	.83	5.10	1.60	5.50	1.52	4.33	1.38	7.53	.98	4.92	1.70	4.26	1.77
**Tension Arousal**	Chinese Instruments	*Erhu*	5.72	1.37	4.90	1.34	4.04	1.48	4.49	1.57	6.68	1.38	5.57	1.51	4.10	1.65	5.59	1.54
		Pipa	6.33	1.35	5.55	1.33	5.01	1.32	4.53	1.27	6.47	1.06	5.65	1.45	4.26	1.89	5.71	1.46
		Dizi	6.65	1.21	5.48	1.40	4.94	1.55	4.33	1.49	5.66	1.41	6.36	1.47	4.30	1.57	5.82	1.36
	Western Instruments	Violin	6.23	1.19	5.01	1.34	4.33	1.51	3.28	1.20	6.85	1.31	5.66	1.47	4.19	1.87	5.42	1.84
		Guitar	5.61	1.23	4.44	1.49	2.79	1.06	2.67	1.11	5.52	1.50	4.88	1.47	2.73	1.24	3.89	1.63
		Flute	5.86	1.54	5.13	1.48	3.34	1.26	3.10	1.06	5.56	1.28	5.21	1.54	3.07	1.50	4.21	1.65

Western Nonmusician Results																	
Affect Dimensions	Culture		Chinese Music								Western Music							
	Affect		Angry		Happy		Peaceful		Sad		Angry		Happy		Peaceful		Sad	
	Descriptive		*M*	*SD*	*M*	*SD*	*M*	*SD*	*M*	*SD*	*M*	*SD*	*M*	*SD*	*M*	*SD*	*M*	*SD*
**Valence**	Chinese Instruments	*Erhu*	6.97	1.33	6.95	.98	4.43	1.79	4.24	1.72	3.83	1.72	6.68	1.39	4.27	2.07	390	1.70
		Pipa	6.87	1.50	7.27	1.08	5.07	1.42	5.16	1.57	4.42	1.53	6.95	1.16	4.47	1.98	4.17	1.58
		Dizi	6.76	1.74	7.13	1.12	4.60	1.75	4.72	1.55	3.99	1.75	6.83	1.45	4.59	1.89	3.96	1.51
	Western Instruments	Violin	6.78	1.46	7.29	1.00	4.52	1.68	4.82	1.68	4.16	1.59	7.23	1.13	4.12	1.98	3.93	1.87
		Guitar	7.37	1.00	7.42	.92	5.32	1.98	5.38	1.91	4.72	2.00	7.34	.90	5.05	2.32	5.02	2.10
		Flute	7.09	1.15	7.28	.98	4.78	1.74	4.49	1.64	4.38	1.48	7.24	.96	4.87	2.19	4.35	1.94
**Tension Arousal**	Chinese Instruments	*Erhu*	5.40	1.59	4.67	1.54	3.89	1.49	4.25	1.75	5.44	1.51	5.23	1.58	4.15	1.95	4.98	1.60
		Pipa	5.70	1.70	5.05	1.73	4.98	1.51	4.44	1.60	5.86	1.17	5.40	1.59	3.88	1.82	5.61	1.54
		Dizi	6.20	1.58	4.90	1.56	4.63	1.63	4.46	1.42	4.94	1.64	5.54	1.69	4.41	1.75	5.35	1.55
	Western Instruments	Violin	5.81	1.63	4.96	1.66	4.27	1.58	3.58	1.43	5.67	1.52	5.33	1.70	4.49	1.74	5.14	1.80
		Guitar	4.98	1.69	3.61	1.48	2.85	1.08	2.76	1.12	4.40	1.55	4.31	1.79	2.66	1.15	3.18	1.11
		Flute	5.28	1.55	4.83	1.62	3.50	1.33	3.65	1.51	5.13	1.26	4.98	1.51	3.47	1.63	4.49	1.72

Chinese Musician Results																		
Affect Dimensions	Culture		Chinese Music								Western Music							
	Affect		Angry		Happy		Peaceful		Sad		Angry		Happy		Peaceful		Sad	
	Descriptive		*M*	*SD*	*M*	*SD*	*M*	*SD*	*M*	*SD*	*M*	*SD*	*M*	*SD*	*M*	*SD*	*M*	*SD*
**Valence**	Chinese Instruments	*Erhu*	6.66	1.18	7.35	1.10	4.08	1.37	2.64	1.16	3.76	1.43	6.76	1.12	3.20	1.02	3.06	1.29
		Pipa	6.86	1.58	7.42	1.11	5.48	1.14	4.04	1.19	4.13	1.17	7.19	1.23	3.65	1.07	3.99	1.27
		Dizi	7.02	1.29	7.53	1.20	5.31	1.37	3.50	1.19	3.64	1.26	7.42	1.23	3.63	.95	3.62	1.04
	Western Instruments	Violin	6.76	1.25	7.44	1.18	4.96	1.41	3.38	1.14	3.92	1.47	7.28	1.26	3.63	.97	3.24	1.38
		Guitar	6.66	1.28	6.92	1.08	4.97	1.11	3.97	1.22	4.13	1.13	7.01	1.19	4.09	1.18	3.57	1.23
		Flute	6.64	1.21	7.09	1.16	4.91	1.27	3.71	1.31	4.01	1.21	7.24	1.17	3.87	1.04	3.68	1.02
**Tension Arousal**	Chinese Instruments	*Erhu*	4.66	1.41	3.61	1.06	4.36	1.40	5.13	1.78	6.51	1.17	4.21	1.30	4.67	1.42	5.53	1.57
		Pipa	5.16	1.67	3.66	1.32	3.93	1.10	4.57	1.54	6.31	.92	4.18	1.56	4.36	1.53	5.62	1.46
		Dizi	5.19	1.68	3.62	1.43	4.43	1.28	5.06	1.62	5.87	1.15	3.98	1.40	4.60	1.36	5.17	1.52
	Western Instruments	Violin	5.23	1.68	3.82	1.42	4.07	1.19	4.61	1.54	6.36	1.16	3.92	1.55	4.52	1.31	5.16	1.67
		Guitar	4.33	1.44	3.50	1.10	3.26	1.20	3.75	1.63	5.27	1.34	3.21	1.25	3.67	1.63	3.95	1.37
		Flute	4.74	1.38	3.91	1.44	3.86	1.20	4.44	1.52	6.08	1.16	3.56	1.26	4.25	1.48	4.48	1.31

Chinese Nonmusician Results																		
Affect Dimensions	Culture		Chinese Music								Western Music							
	Affect		Angry		Happy		Peaceful		Sad		Angry		Happy		Peaceful		Sad	
	Descriptive		*M*	*SD*	*M*	*SD*	*M*	*SD*	*M*	*SD*	*M*	*SD*	*M*	*SD*	*M*	*SD*	*M*	*SD*
**Valence**	Chinese Instruments	*Erhu*	6.95	.97	7.08	.89	3.23	.93	2.57	.89	3.35	1.09	7.09	.80	2.90	.78	2.98	.97
		Pipa	7.32	.95	7.27	.80	4.95	1.02	3.75	1.12	4.11	.97	7.38	.70	3.58	1.03	4.04	1.07
		Dizi	7.31	1.13	7.65	.57	4.33	1.23	3.37	1.16	3.19	.95	7.58	.71	3.56	.94	3.50	.98
	Western Instruments	Violin	7.09	.80	7.46	.63	3.74	.94	3.32	.98	3.51	1.18	7.61	.70	3.29	1.03	3.03	.96
		Guitar	7.02	.84	6.97	.85	4.29	1.23	3.95	1.25	4.07	1.03	7.06	.88	3.98	1.08	3.51	1.30
		Flute	6.95	.91	7.21	1.03	3.92	1.03	3.45	1.15	4.01	1.03	7.36	.69	3.75	1.04	3.42	1.04
**Te1.nsion Arousal**	Chinese Instruments	*Erhu*	4.71	1.45	4.18	1.25	4.67	1.18	4.85	1.34	5.77	1.10	4.22	1.22	4.70	1.41	5.38	1.11
		Pipa	5.09	1.63	4.23	1.28	4.26	1.20	4.14	1.21	5.39	1.02	3.99	1.35	4.07	1.38	5.00	1.18
		Dizi	4.90	1.62	4.10	1.16	4.47	1.43	4.75	1.13	5.12	1.29	4.12	1.37	4.50	1.24	5.36	1.24
	Western Instruments	Violin	5.14	1.63	4.23	1.21	4.40	1.27	4.22	1.27	5.83	1.07	4.12	1.42	4.54	1.48	4.99	1.35
		Guitar	4.41	1.55	3.51	1.02	3.45	1.15	3.43	1.12	4.71	1.13	3.65	1.12	3.17	1.22	3.47	1.09
		Flute	4.76	1.39	4.11	1.24	4.13	1.12	4.12	1.09	5.28	.92	3.87	1.28	3.90	1.19	4.57	1.36

Western Musician Results																	
Affect Dimensions	Culture	Chinese Music								Western Music							
	Affect	Angry		Happy		Peaceful		Sad		Angry		Happy		Peaceful		Sad	
	Descriptive	*M*	*SD*	*M*	*SD*	*M*	*SD*	*M*	*SD*	*M*	*SD*	*M*	*SD*	*M*	*SD*	*M*	*SD*
**Energy Arousal**	Chinese Instruments *Erhu*	7.34	1.44	6.94	.97	3.90	1.07	4.67	1.41	6.24	1.29	7.00	1.21	3.53	1.43	3.97	1.33
	Pipa	7.90	1.01	7.57	.78	5.36	1.19	4.02	1.45	5.92	1.38	7.69	.76	3.23	1.52	4.95	1.46
	Dizi	8.02	.92	7.59	.85	4.67	1.14	4.48	1.34	4.66	1.33	8.14	.79	4.04	1.26	4.43	1.34
	Western Instruments Violin	7.62	.97	7.56	.89	4.21	1.26	3.44	1.07	6.34	1.06	7.76	.88	3.30	1.16	4.16	1.40
	Guitar	7.49	.96	6.84	1.09	3.30	1.10	3.03	.88	5.25	1.37	7.13	1.15	2.69	1.13	3.31	1.52
	Flute	7.66	1.03	7.41	.96	3.52	.83	3.51	1.07	5.57	1.26	7.57	.96	3.23	1.33	3.79	1.05
**Preference**	Chinese Instruments *Erhu*	5.71	2.04	6.61	1.85	6.35	1.83	6.59	1.95	6.17	1.59	5.42	2.02	6.18	1.80	5.84	2.02
	Pipa	5.99	2.12	6.16	1.88	5.96	1.98	5.71	2.07	5.84	1.69	5.40	1.99	6.00	1.68	5.43	2.12
	Dizi	4.62	2.02	6.30	1.97	5.43	1.98	5.52	1.87	5.53	1.75	5.01	2.12	5.80	1.71	5.07	2.01
	Western Instruments Violin	5.60	1.89	6.28	1.76	5.68	1.84	6.01	1.71	6.67	1.46	6.18	1.92	6.26	1.75	6.14	2.17
	Guitar	5.80	1.90	6.59	1.92	6.47	1.94	6.55	1.79	6.37	1.85	6.01	2.87	6.85	1.74	6.92	1.52
	Flute	5.63	1.87	6.23	1.70	5.92	1.76	5.77	1.83	5.90	1.66	5.90	1.91	6.55	1.70	6.28	1.97

Western Nonmusician Results																		
Affect Dimensions	Culture		Chinese Music								Western Music							
	Affect		Angry		Happy		Peaceful		Sad		Angry		Happy		Peaceful		Sad	
	Descriptive		*M*	*SD*	*M*	*SD*	*M*	*SD*	*M*	*SD*	*M*	*SD*	*M*	*SD*	*M*	*SD*	*M*	*SD*
**Energy Arousal**	Chinese Instruments	*Erhu*	7.38	.98	6.94	1.21	3.72	1.53	3.71	1.27	4.60	1.80	6.80	1.17	3.32	1.69	3.52	1.49
		Pipa	7.65	.93	7.39	.99	5.21	1.59	4.73	1.54	5.25	1.53	7.28	1.12	3.24	1.76	4.80	1.85
		Dizi	7.86	.97	7.29	.99	4.82	1.71	4.17	1.52	3.93	1.40	7.44	1.16	3.90	1.65	4.43	1.79
	Western Instruments	Violin	7.54	.95	7.53	.83	4.03	1.39	3.52	1.28	4.96	1.50	7.46	.96	3.54	1.61	4.16	1.97
		Guitar	7.49	.94	6.94	1.13	3.77	1.66	3.57	1.61	4.49	1.48	7.18	.93	3.12	1.58	3.80	1.78
		Flute	7.35	1.17	7.45	.91	3.67	1.54	3.58	1.55	4.71	1.36	7.20	.94	3.58	1.63	3.89	1.73
**Preference**	Chinese Instruments	*Erhu*	5.32	1.71	6.30	1.35	5.43	1.74	5.27	2.05	4.93	2.03	5.48	1.46	5.65	2.15	5.08	2.01
		Pipa	5.47	1.82	6.26	1.58	5.28	1.70	5.05	1.89	5.27	1.65	5.57	1.61	5.52	1.95	4.69	2.12
		Dizi	4.78	1.81	6.05	1.58	4.75	2.00	4.82	1.73	5.00	2.08	5.27	1.78	5.45	1.82	4.30	2.00
	Western Instruments	Violin	5.60	1.64	6.45	1.43	5.25	1.97	5.56	1.59	5.35	1.71	6.02	1.40	5.57	1.94	5.40	1.96
		Guitar	6.22	1.44	7.16	1.22	6.31	1.70	6.46	1.73	6.02	1.77	6.69	1.39	6.67	1.81	6.75	1.80
		Flute	5.69	1.54	6.18	1.27	5.50	2.03	5.43	1.69	4.78	1.60	6.18	1.47	6.04	1.97	5.58	2.10

Chinese Musician Results																		
Affect Dimensions	Culture		Chinese Music								Western Music							
	Affect		Angry		Happy		Peaceful		Sad		Angry		Happy		Peaceful		Sad	
	Descriptive		*M*	*SD*	*M*	*SD*	*M*	*SD*	*M*	*SD*	*M*	*SD*	*M*	*SD*	*M*	*SD*	*M*	*SD*
**Energy Arousal**	Chinese Instruments	*Erhu*	6.69	1.06	7.05	.87	4.22	1.01	3.50	1.53	5.74	1.50	6.62	.97	3.21	.92	3.71	1.17
		Pipa	7.56	.99	7.32	.82	5.44	.95	3.98	1.11	5.95	1.19	7.15	.87	3.36	.92	5.02	1.22
		Dizi	7.59	.80	7.48	.76	5.43	1.20	3.94	1.30	4.61	1.13	7.55	.84	4.03	.91	4.10	1.15
	Western Instruments	Violin	7.29	.95	7.27	.78	4.55	1.07	3.37	.96	6.10	1.39	7.58	.75	3.68	1.17	4.29	1.31
		Guitar	6.85	.98	6.55	.93	3.96	.92	3.19	.73	4.89	1.20	6.66	.93	3.28	1.10	3.47	1.03
		Flute	6.80	1.11	6.94	.91	4.23	.92	3.35	.99	5.27	1.19	7.13	.91	3.65	.94	4.24	.98
**Preference**	Chinese Instruments	*Erhu*	5.95	1.60	6.62	1.09	6.11	1.42	6.88	1.44	6.33	1.41	5.83	1.48	5.49	1.51	5.66	1.52
		Pipa	6.79	1.31	6.92	1.22	6.40	1.36	5.99	1.48	5.24	1.67	6.09	1.44	5.90	1.30	5.30	1.87
		Dizi	5.98	1.46	6.80	1.21	5.69	1.31	5.31	1.60	5.05	1.73	6.23	1.54	5.49	1.48	4.74	2.00
	Western Instruments	Violin	6.34	1.31	6.70	1.31	5.77	1.28	5.94	1.30	6.79	1.49	6.83	1.39	5.99	1.36	6.48	1.62
		Guitar	6.44	1.12	6.66	1.17	6.71	1.01	6.53	1.33	6.09	1.64	6.95	1.03	6.51	1.24	6.64	1.61
		Flute	5.62	1.44	5.97	1.22	6.17	1.32	5.74	1.56	5.68	1.57	6.52	1.22	5.99	1.17	5.91	1.55

Chinese Nonmusician Results																		
Affect Dimensions	Culture		Chinese Music								Western Music							
	Affect		Angry		Happy		Peaceful		Sad		Angry		Happy		Peaceful		Sad	
	Descriptive		*M*	*SD*	*M*	*SD*	*M*	*SD*	*M*	*SD*	*M*	*SD*	*M*	*SD*	*M*	*SD*	*M*	*SD*
**Energy Arousal**	Chinese Instruments	*Erhu*	7.01	.82	6.67	1.06	3.70	1.01	3.33	.86	4.90	1.33	6.86	.68	3.34	.84	3.94	1.08
		Pipa	7.50	.63	7.07	.72	5.03	1.21	4.16	.98	5.25	1.08	7.12	.76	3.40	.97	4.82	1.06
		Dizi	7.47	.76	7.10	.65	4.81	1.38	3.99	1.19	4.43	1.19	7.33	.75	3.95	.93	4.49	1.10
	Western Instruments	Violin	7.12	.84	7.11	.83	4.03	.84	3.58	.80	5.17	1.40	7.33	.72	3.93	.99	4.10	1.28
		Guitar	6.76	1.16	6.47	.90	3.84	.96	3.60	.94	4.52	1.10	6.52	.94	3.68	.96	3.85	1.34
		Flute	6.83	.95	6.78	.80	4.02	.92	3.50	.79	5.11	1.16	7.07	.77	3.93	.97	4.19	1.09
**Preference**	Chinese Instruments	*Erhu*	6.05	1.25	5.89	.97	4.99	1.03	4.99	1.53	5.19	1.26	5.92	1.11	4.76	1.21	5.09	1.48
		Pipa	6.51	1.07	6.00	1.08	5.73	1.31	5.69	1.25	5.39	1.18	6.21	.99	5.17	1.31	5.30	1.45
		Dizi	5.82	1.43	6.08	.83	5.12	1.42	4.79	1.53	5.27	1.18	6.07	1.25	5.12	1.28	5.02	1.29
	Western Instruments	Violin	6.12	1.28	6.35	.81	4.92	1.24	5.38	1.16	5.89	1.44	6.61	1.09	5.39	1.30	5.56	1.52
		Guitar	6.59	.98	6.62	.88	5.96	1.22	6.01	.94	5.95	1.03	6.64	.87	6.30	1.31	6.63	1.20
		Flute	5.93	1.29	6.14	.74	5.31	.97	5.17	1.17	5.42	.90	6.36	.94	5.59	1.15	5.76	1.44

Familiarity Results																
Culture	Chinese Music								Western Music							
Affect	Angry		Happy		Peaceful		Sad		Angry		Happy		Peaceful		Sad	
Descriptive	*M*	*SD*	*M*	*SD*	*M*	*SD*	*M*	*SD*	*M*	*SD*	*M*	*SD*	*M*	*SD*	*M*	*SD*
Western Musicians	2.85	2.24	3.34	2.50	2.77	2.08	2.84	2.41	4.45	2.97	6.30	3.11	8.04	1.90	6.00	2.88
Western Nonmusicians	2.60	1.76	3.46	2.49	2.72	2.16	2.96	2.41	3.18	2.27	3.22	2.40	5.85	2.86	3.50	2.76
Chinese Musicians	8.71	.564	8.09	1.51	7.05	2.42	7.89	2.13	7.81	1.77	6.78	2.76	5.08	3.03	5.46	2.88
Chinese Nonmusicians	8.60	.87	7.04	1.37	4.33	2.19	5.35	2.23	6.78	2.16	5.72	2.59	4.87	2.24	5.17	2.82

3. Tables for the 5-way ANOVA and 4-way ANOVAs for each emotion.

Table S2. The five-way ANOVA results for valence, tension arousal, energy arousal, preference and familiarity.

Effect	Valence				Tension				Energy				Preference			
	F	*p*	*df*	η_p^2	*F*	*p*	*df*	η_p^2	*F*	*p*	*df*	η_p^2	*F*	*p*	*df*	η_p^2
LG	7.33	<.001	3, 156	.12	4.96	.003	3,156	.09	1.43	.24	3, 156	.03	1.86	.14	3, 156	.04
ICU	22.28	<.001	1, 156	.13	218.00	<.001	1, 156	.58	95.99	<.001	1, 156	.38	99.48	<.001	1, 156	.39
ICA	68.84	<.001	1.89, 294	.31	72.57	<.001	1.84, 286.65	.32	5.56	.005	1.90, 296.26	.03	59.92	<.001	1.81, 282.36	.28
MC	402.24	<.001	1, 156	.72	66.21	<.001	1, 156	.29	290.57	<.001	1, 156	.65	4.61	.033	1, 156	.03
ME	485.27	<.001	1.44, 224.13	.76	68.59	<.001	1.38, 214.52	.31	931.59	<.001	1.40, 281.72	.86	24.51	<.001	1.94, 303.32	.14
LG X ICU	4.13	.007	3, 156	.07	5.47	.001	3, 156	.10	4.01	.009	3, 156	.07	2.23	.087	3, 156	.04
LG X ICA	2.82	.011	5.65, 294	.05	1.15	.335	5.50, 286.22	.02	2.38	.032	5.70, 296.23	.04	3.82	.002	5.43, 282.36	.07
LG X MC	2.01	.115	3, 156	.04	2.82	.04	3, 156	.05	4.69	.004	3, 156	.08	1.23	.302	3, 156	.02
LG X ME	5.89	<.001	4.31, 224.13	.10	8.96	<.001	4.11, 213.73	.15	3.27	.011	4.21, 218.72	.06	4.76	<.001	5.83, 303.32	.08
ICU X ICA	6.50	.002	1.81, 281.86	.04	135.20	<.001	193, 300.78	.46	173.86	<.001	1.84, 286.29	.53	23.25	<.001	1.97, 307.48	.08
ICU X MC	22.38	<.001	1, 156	.13	.76	.39	1, 156	.01	85.97	<.001	1, 156	.36	104.71	<.001	1, 156	.40
ICU X ME	2.04	.11	2.85, 444.68	.01	63.96	<.001	2.90, 452.02	.29	41.01	<.001	2.79,435.73	.21	12.97	<.001	2.94, 457.90	.08
ICA X MC	.95	.39	2, 312	.01	37.49	<.001	2, 312	.20	18.15	<.001	2, 312	.10	7.15	.001	2, 312	.04
ICA X ME	32.78	<.001	5.81, 906.59	.17	8.26	<.001	5.37, 837.59	.05	22.83	<.001	5.48, 854.55	.13	14.71	<.001	5.63, 878.43	.09
MC X ME	246.15	<.001	1.97, 307.62	.61	19.38	<.001	2.05, 320.12	.11	331.87	<.001	2.14,333.49	.68	5.70	.001	2.66, 414.21	.04
LG X ICU X ICA	9.35	<.001	5.42, 281.86	.15	8.4	<.001	5.78, 300.78	.14	.85	.46	5.51, 286.63	.02	4.49	<.001	5.91, 307.48	.08
LG X ICU X MC	.07	.98	3, 156	.001	3.51	.017	3, 156	.06	.80	.50	3, 156	.02	8.93	<.001	3, 156	.15

Effect	Valence				Tension				Energy				Preference			
	F	*p*	*df*	η_p^2	*F*	*p*	*df*	η_p^2	*F*	*p*	*df*	η_p^2	*F*	*p*	*df*	η_p^2
LG X ICU X ME	1.3	.24	8.55, 444.68	.02	1.73	.081	8.75, 454.88	.03	6.62		8.38, 435.73	.11	.79	.62	8.80, 457.90	.02
LG X ICA X MC	.95	.67	6, 312	.01	2.4	.026	6, 312	.05	.61	.72	6, 312	.01	2.14	.049	6, 312	.04
LG X ICA X ME	1.88	.016	17.43, 906.59	.04	1.77	.032	15.93, 828.38	.03	2.26	.003	16.43, 854.55	.04	2.25	.003	16.89, 878.43	.04
LG X MC X ME	7.1	<.001	5.92, 307.62	.12	8.59	<.001	6.07, 315.719	.14	5.9	<.001	6.41, 333.49	.09	5.90	<.001	7.97, 414.21	.04
ICU X ICA X MC	17.81	<.001	1.97, 307.09	.10	1.26	.29	2, 312	.01	11.4	<.001	2, 312	.07	3.96	.02	1.98, 309.52	.03
ICU X ICA X ME	5.99	<.001	5.79, 903.87	.04	3.47	.002	6, 936	.02	11.79	<.001	5.79, 903.08	.07	17.68	<.001	5.89, 918.70	.10
ICU X MC X ME	9.84	<.001	3, 468	..06	5.02	.002	3, 468	.03	14.53	<.001	2.89, 450	.09	6.79	<.001	2.71, 423.35	.04
ICA X MC X ME	6.02	<.001	5.58, 871.08	.04	4.91	<.001	5.76, 898.65	.03	26.84	<.001	5.83, 908.83	.15	5.26	<.001	6, 936	.03
LG X ICU X ICA X MC	1.49	.18	6, 312	.03	1.59	.15	6, 312	.03	1.39	.22	6, 312	.03	.92	.48	5.95, 309.52	.02
LG X ICU X ICA X ME	1.93	.013	17.38, 903.87	.04	1.80	.021	18. 936	.03	1.53	.075	17.31, 900.15	.03	1.55	.068	17.67, 918.70	.03
LG X ICU X MC X ME	4.26	<.001	9, 468	.08	1.38	.21	9, 468	.03	1.99	.041	8.65, 449.53	.04	2.79	.005	8.14, 423.35	.05
LG X ICA X MC X ME	1.7	.038	16.75, 871.08	.03	2.20	.003	17.28, 898.65	.04	3.23	<.001	17.84, 927.69	.06	2.05	.006	18, 936	.04
ICU X ICA X MC X ME	5.65	<.001	5.89, 919.16	.04	15.53	<.001	6, 936	.09	38.87	<.001	5.74, 894.85	.20	4.44	<.001	5.90, 919.59	.03
LG X ICU X ICA X MC X ME	1.65	.044	17.68, 919.16	.03	1.67	.04	18, 936	.03	2.10	.005	17.21, 894.85	.04	2.84	<.001	17.68, 919.59	.05

Effect	Familiarity			
	F	*p*	*df*	η_p^2
LG	50.82	<.001	3, 156	.49
MC	13.69	<.001	1, 156	.08
ME	9.76	<.001	3, 468	.06
LG X MC	69.97	<.001	3, 156	.57
LG X ME	22.35	<.001	9, 468	.30
MC X ME	15.95	<.001	2.93, 457.51	.09
LG X MC X ME	5.56	<.001	8.80, 457.51	.10

Table S3. Four-way ANOVAs for each intended emotion on valence, tension arousal, energy arousal and preference.

Effect	Valence															
	Angry				Happy				Peaceful				Sad			
	F	*p*	*df*	η_p^2	*F*	*p*	*df*	η_p^2	*F*	*p*	*df*	η_p^2	*F*	*p*	*df*	η_p^2
**MAIN**																
ICA	17.35	<.001	1.97, 307.15	.10	6.28	.02	1.98, 308.92	.04	72.99	<.001	1.96, 306.16	.32	68.53	<.001	2, 312	.31
ICU	16.95	<.001	1, 156	.10	7.91	.06	1, 156	.05	15.22	<.001	1, 156	.09	15.27	<.001	1, 156	.09
MC	472.97	<.001	1, 156	.75	2.28	.13	1, 156	.01	74.99	<.001	1, 156	.33	41.01	<.001	1, 156	.21
**2-WAY**																
ICA X ICU	4.54	.012	1.96, 305.76	.03	24.45	<.001	2, 312	.14	2.69	.069	2, 312	.02	1.22	.30	2, 312	.01
ICA X MC	6.15	.02	2, 156	.04	2.46	.087	2, 312	.02	9.67	<.001	2, 312	.06	.75	.47	2, 312	.01
ICU X MC	13.49	<.001	1, 156	.08	19.90	<.001	1, 156	.11	19.98	<.001	1, 156	.11	3.54	.062	1, 156	.02
**3-WAY**																
ICU X ICA X MC	6.60	.002	2, 312	.04	.49	.61	2, 312	.003	16.31	<.001	2, 312	.10	5.89	.003	2, 312	.04
**GROUP**																
LG	1.21	.301	3, 156	.02	.32	.81	3, 156	.006	5.35	.02	3, 156	.09	13.81	<.001	3, 156	.21
ICA X LG	.98	.44	5.91, 307.15	.02	6.28	.002	1.98, 308.92	.07	1.44	.20	5.89, 306.16	.03	3.59	.002	6, 312	.07
ICU X LG	3.19	.025	3, 156	.06	9.48	<.001	3, 156	.15	.83	.48	3, 156	.02	1.55	.20	3, 156	.03
MC X LG	1.22	.31	3, 156	.02	1.47	.23	3, 156	.03	8.80	<.001	3, 156	.15	17.82	<.001	3, 156	.26
ICA X ICU X LG	2.96	.008	5.88, 305.78	.05	4.24	<.001	6, 312	.08	5.62	<.001	6, 312	.10	6.23	<.001	5.53, 287.55	.11
ICA X MC X LG	1.46	.19	6, 312	.03	1.42	.21	6, 312	.03	1.63	.14	6, 312	.03	1.35	.23	6, 312	.03
ICU X MC X LG	1.96	.12	3, 156	.04	.78	.51	3, 156	.02	1.95	.12	3, 156	.04	6.45	<.001	3, 156	.11
ICA X ICU X MC X LG	2.59	.019	6, 312	.05	.25	.96	6, 312	.005	1.05	.40	6, 312	.02	2.12	.051	6, 312	.04

Effect	Tension Arousal															
	Angry				Happy				Peaceful				Sad			
	F	*p*	*df*	η_p^2	*F*	*p*	*df*	η_p^2	*F*	*p*	*df*	η_p^2	*F*	*p*	*df*	η_p^2
**MAIN**																
ICA	28.02	<.001	2, 312	.15	21.89	<.001	1.90, 296.01	.12	45.28	<.001	1.86, 290.00	.23	49.58	<.001	1.84, 287.90	.24
ICU	31.36	<.001	1, 156	.17	55.12	<.001	1, 156	.26	210.12	<.001	1, 156	.57	246.28	<.001	1, 156	.61
MC	8.39	.004	1, 156	.05	21.02	<.001	1, 156	.12	.68	.41	1, 156	.004	135.04	<.001	1, 156	.46
**2-WAY**																
ICA X ICU	72.39	<.001	2, 312	.32	39.94	<.001	2, 312	.20	68.32	<.001	1.97, 307.64	.31	46.08	<.001	2, 312	.23
ICA X MC	37.08	<.001	2, 312	.19	2.29	.10	1.98. 309.25	.01	7.95	<.001	1.99, 309.99	.05	4.78	.009	2, 312	.03
ICU X MC	.002	.96	1, 156	.00	10.46	.001	1, 156	.06	4.90	.028	1, 156	.03	.63	.43	1, 156	.004
**3-WAY**																
ICU X ICA X MC	20.58	<.001	2, 312	.12	5.76	.004	2, 312	.04	2.17	.12	2, 312	.01	20.43	<.001	2, 312	.12
**GROUP**																
LG	10.57	<.001	3, 156	.17	16.89	<.001	3, 156	.25	.75	.52	3, 156	.01	1.56	.20	3, 156	.03
ICA X LG	.74	.62	6, 312	.01	2.24	.043	5.69, 296.01	.04	.91	.49	5.58, 290.00	.02	2.16	.05	5.54, 287.90	.04
ICU X LG	1.04	.38	3, 156	.02	2.70	.048	3, 156	.05	7.77	<.001	3, 156	.13	4.23	.007	3, 156	.08
MC X LG	6.85	<.001	3, 156	.12	5.44	.001	3, 156	.10	5.37	.002	3, 156	.09	9.86	<.001	3, 156	.16
ICA X ICU X LG	1.29	.26	6, 312	.02	3.95	.001	6, 312	.07	8.56	<.001	5.92, 307.64	.14	3.10	.006	6, 312	.06
ICA X MC X LG	4.07	.001	6, 312	.07	.95	.46	5.95, 309.25	.02	2.19	.05	5.96, 309.99	.04	1.72	.12	6, 312	.03
ICU X MC X LG	.21	.89	3, 156	.004	4.10	.008	3, 156	.07	1.64	.18	3, 156	.03	1.87	.14	3, 156	.04
ICA X ICU X MC X LG	1.76	.11	6, 312	.03	1.26	.28	6, 312	.02	1.83	.09	6, 312	.03	1.76	.11	6, 312	.03

Effect	Energy Arousal															
	Angry				Happy				Peaceful				Sad			
	F	*p*	*df*	η_p^2	*F*	*p*	*df*	η_p^2	*F*	*p*	*df*	η_p^2	*F*	*p*	*df*	η_p^2
**MAIN**																
ICA	14.83	<.001	1.94, 303.05	.09	32.37	<.001	2, 312	.17	20.16	<.001	2, 312	.11	6.49	.002	1.92, 298.87	.04
ICU	7.70	.006	1, 156	.05	5.31	.023	1, 156	.03	88.62	<.001	1, 156	.36	108.77	<.001	1, 156	.41
MC	474.11	<.001	1, 156	.75	7.21	.008	1, 156	.04	197.12	<.001	1, 156	.56	52.07	<.001	1, 156	.25
**2-WAY**																
ICA X ICU	58.35	<.001	1.91, 298.19	.27	130.97	<.001	2, 312	.46	89.10	<.001	2, 312	.36	55.35	<.001	2, 312	.26
ICA X MC	46.48	<.001	2, 312	.23	2.25	.11	2, 312	.01	36.05	<.001	2, 312	.19	1.07	.34	2, 312	.007
ICU X MC	24.35	<.001	1, 156	.14	3.81	.053	1, 156	.02	92.27	<.001	1, 156	.37	7.14	.008	1, 156	.04
**3-WAY**																
ICU X ICA X MC	54.95	<.001	2, 312	.26	5.52	.004	2, 312	.03	40.93		2, 312	.21	21.20	<.001	2, 312	.12
**GROUP**																
LG	8.35	<.001	3, 156	.14	3.57	.016	3, 156	.06	1.13	.34	3, 156	.02	.22	.88	3, 156	.004
ICA X LG	1.96	.07	5.83, 303.05	.04	2.55	.02	6, 312	.05	1.35	.24	6, 312	.03	3.09	.007	5.75, 298.87	.06
ICU X LG	1.42	.24	3, 156	.03	3.33	.021	3, 156	.06	7.84	<.001	3, 156	.13	6.37	<.001	3, 156	.11
MC X LG	6.16	.001	3, 156	.11	3.15	.027	3, 156	.06	4.20	.007	3, 156	.08	3.08	.029	3, 156	.06
ICA X ICU X LG	1.70	.13	5.74, 298.19	.03	1.36	.23	6, 312	.03	.73	.63	6, 312	.01	1.40	.21	6, 312	.03
ICA X MC X LG	4.76	<.001	6, 312	.08	.93	.47	6, 312	.02	1.36	.23	6, 312	.03	2.07	.057	6, 312	.04
ICU X MC X LG	.59	.62	3, 156	.01	3.44	.018	3, 156	.06	1.49	.22	3, 156	.03	1.87	.14	3, 156	.04
ICA X ICU X MC X LG	.79	.58	6, 312	.02	1.55	.16	6, 312	.03	.88	.51	6, 312	.02	4.07	.001	6, 312	.07

Effect	Preference															
	Angry				Happy				Peaceful				Sad			
	F	*p*	*df*	η_p^2	*F*	*p*	*df*	η_p^2	*F*	*p*	*df*	η_p^2	*F*	*p*	*df*	η_p^2
**MAIN**																
ICA	55.10	<.001	2, 312	.26	13.92	<.001	1.75, 272.31	.08	38.38	<.001	1.88, 293.67	.20	44.63	<.001	1.87, 291.15	.22
ICU	45.29	<.001	1, 156	.23	44.45	<.001	1, 156	.22	57.74	<.001	1, 156	.27	100.24	<.001	1, 156	.39
MC	2.79	<.001	1, 156	.02	30.95	<.001	1, 156	.17	2.23	<.001	1, 156	.01	.55	.46	1, 156	.003
**2-WAY**																
ICA X ICU	.11	.89	2, 312	.00	7.09	.001	2, 312	.04	25.30	<.001	1.97, 307.50	.14	41.06	<.001	2, 312	.21
ICA X MC	14.16	<.001	2, 312	.08	.67	.51	2, 312	.004	4.19	.016	2, 312	.03	1.79	.17	2, 312	.01
ICU X MC	10.26	.002	1, 156	.06	56.68	<.001	1, 156	.27	28.30	<.001	1, 156	.15	50.3	<.001	1, 156	.24
**3-WAY**																
ICU X ICA X MC	10.97	<.001	1.97, 307.87	.07	3.47	.032	2, 312	.02	4.35	.014	2, 312	.03	.14	.87	2, 312	.001
**GROUP**																
LG	2.37	.07	3, 156	.04	1.44	.23	3, 156	.03	3.28	.023	3, 156	.06	2.55	.058	3, 156	.05
ICA X LG	4.26	<.001	6, 312	.08	1.76	.12	5.24, 272.31	.03	1.95	.078	5.64, 293.67	.04	4.11	.001	5.60, 291.15	.07
ICU X LG	.91	.44	3, 156	.02	2.15	.096	3, 156	.04	.81	.49	3, 156	.02	2.28	.08	3, 156	.04
MC X LG	5.14	.002	3, 156	.09	9.61	<.001	3, 156	.16	2.99	.033	3, 156	.05	1.41	.24	3, 156	.03
ICA X ICU X LG	3.01	.007	6, 312	.06	3.49	.002	6, 312	.06	2.70	.015	5.91, 307.50	.05	2.63	.017	6, 312	.05
ICA X MC X LG	5.30	<.001	6, 312	.09	1.07	.38	6, 312	.02	.57	.75	6, 312	.01	.71	.64	6, 312	.01
ICU X MC X LG	7.68	<.001	3, 156	.13	6.75	<.001	3, 156	.12	3.38	.02	3, 156	.06	2.33	.076	3, 156	.04
ICA X ICU X MC X LG	4.85	<.001	5.92, 307.87	.09	1.07	.38	6, 312	.02	.64	.70	6, 312	.01	2.83	.011	6, 312	.05

4. Table S4. The top five important acoustic features based on VIP scores from the PLSR models for valence, tension arousal and energy arousal.

	Western Participants	Chinese Participants
Valence	Spectral Flux	Spectral Flux
	Effective Duration	Effective Duration
	Attack Time	Attack Time
	Frame Energy ERB IQR	Event Density
	Event Density	Frame Energy ERB IQR
Tension Arousal	Spectral Flux	Frequency Modulation
	Spectral Flatness	Frame Energy of ERB
	Decrease Time	Event Density SD
	Spectral Kurtosis	Spectral Kurtosis
	Spectral Skewness	Spectral Brightness
Energy Arousal	Spectral Flux	Spectral Flux
	Event Density	Effective Duration
	Effective Duration	Event Density
	Frame Energy ERB IQR	Attack Time
	Attack Time	Frame Energy ERB IQR
